# Unconscious Perception of Vernier Offsets

**DOI:** 10.1162/opmi_a_00145

**Published:** 2024-06-04

**Authors:** Pietro Amerio, Matthias Michel, Stephan Goerttler, Megan A. K. Peters, Axel Cleeremans

**Affiliations:** Consciousness, Cognition & Computation Group, Center for Research in Cognition & Neurosciences, ULB Neuroscience Institute, Université libre de Bruxelles; Center for Mind, Brain and Consciousness, New York University; Department of Cognitive Sciences, University of California Irvine

**Keywords:** unconscious perception, consciousness, tachistoscope, bias-free task, observer model

## Abstract

The comparison between conscious and unconscious perception is a cornerstone of consciousness science. However, most studies reporting above-chance discrimination of unseen stimuli do not control for criterion biases when assessing awareness. We tested whether observers can discriminate subjectively invisible offsets of Vernier stimuli when visibility is probed using a bias-free task. To reduce visibility, stimuli were either backward masked or presented for very brief durations (1–3 milliseconds) using a modern-day Tachistoscope. We found some behavioral indicators of perception without awareness, and yet, no conclusive evidence thereof. To seek more decisive proof, we simulated a series of Bayesian observer models, including some that produce visibility judgements alongside type-1 judgements. Our data are best accounted for by observers with slightly suboptimal conscious access to sensory evidence. Overall, the stimuli and visibility manipulations employed here induced mild instances of blindsight-like behavior, making them attractive candidates for future investigation of this phenomenon.

## INTRODUCTION

Unconscious vision is characterized as involving above-chance performance in a visual task while participants fail to consciously see the stimuli (Breitmeyer, 2015; Merikle et al., 2001; Ramsøy & Overgaard, 2004). Comparing conscious and unconscious visual processing is a key approach in consciousness studies: the hope is that it will reveal the neural correlates of consciousness, as well as the psychological functions associated with it (Baars, 1995; Hannula et al., 2005). The recent rise in skepticism about unconscious vision, both in healthy volunteers (Balsdon & Clifford, 2018; Peters & Lau, 2015; Phillips, 2018) and in blindsight patients (Michel & Lau, 2021; Phillips, 2021) thus constitutes a threat to one of the methodological and conceptual cornerstones of consciousness research (LeDoux et al., 2020; Peters, Kentridge, et al., 2017).

A major motivation for skepticism is the ‘criterion problem’ (Cheesman & Merikle, 1984; Eriksen, 1960; Goldiamond, 1958; Holender, 1986; Merikle, 1982; Phillips, 2016). The argument goes as follows: an observer’s report that she failed to see a stimulus can be interpreted in two ways. In the first instance, the observer indeed failed to consciously perceive the stimulus. In the second instance, the strength of the sensory signal associated with the stimulus fell below a (potentially conservatively biased) criterion for reporting the stimulus as ‘seen’ (Macmillan & Creelman, 2004; Phillips, 2016). This latter interpretation crucially entails that what scientists routinely take as evidence for unconscious perception could simply result from participants adopting an overly conservative criterion and hence failing to report seeing stimuli that nevertheless elicited weak conscious perceptual signals.

Researchers have recently attempted to solve this problem by relying on 2-Interval Forced-Choice (2IFC) tasks to collect subjective ratings (de Gardelle & Mamassian, 2014; Knotts et al., 2018; Mamassian, 2020; Peters, Fesi, et al., 2017; Peters & Lau, 2015). Instead of asking for a free visibility or confidence judgment, which would involve setting a criterion for selecting the ‘confident’ or ‘seen’ response, in these paradigms, participants first perform a discrimination task in two successive intervals and subsequently express a forced-choice judgment comparing the extent of their introspective access to perceptual information in the two intervals. For example, Peters and Lau (2015) had participants discriminate the orientation of a masked Gabor patch in each interval. Participants then had to bet on the interval in which they felt the most confident in their discrimination decision. Crucially, only one interval contained a Gabor patch, and participants were not informed that the other interval would always be empty. This is the key element to using 2IFC-based tasks to measure subjective visibility: if participants fail to bet on the stimulus-present interval above chance, but are nevertheless able to discriminate the stimulus, the latter was perceived unconsciously. Since participants are forced to choose and the task does not bias participants to select either one of the intervals, 2IFC-based paradigms are relatively free from response biases (Green & Swets, 1966; Macmillan & Creelman, 2004; Mamassian, 2020; although, see Yeshurun et al., 2008). Hence, using 2IFC-based paradigms rather than subjective ratings makes it possible to sidestep the criterion problem and gather robust evidence for the existence of unconscious perception.

Interestingly, these 2IFC-based tasks designed with a stimulus-absent interval thus far produced no evidence of unconscious visual perception (Peters, Fesi, et al., 2017; Peters & Lau, 2015). Rajananda et al. (2020) first raised the concern that participants could succeed in betting on the interval that contained the stimulus, even if they remained unaware of its task-relevant feature (i.e., the orientation of the Gabor patch, in the example above). Indeed, being able to tell that an interval contained “something” (vs. nothing) does not imply that one is aware of the feature of the stimulus that is relevant for the discrimination task (Michel, 2023; Rajananda et al., 2020). It could be the case that the mere presence of some indeterminate shape can be consciously detected while the *orientation* of the Gabor patch itself remains unconscious (Breitmeyer, 1984; Breitmeyer et al., 2006; Jimenez et al., 2019; Kahneman, 1968; Koivisto & Neuvonen, 2020). As such, Peters & Lau’s paradigm allows experimenters to evaluate the extent of perceptual processing in cases in which the observer is fully unaware of the stimulus. However, demonstrating unconscious perception only requires showing above-chance discrimination of a feature of the stimulus when participants are unaware of *that* feature—not complete unawareness of the stimulus (Michel, 2023).

Rajananda et al. (2020) adapted the 2IFC-based design such that a stimulus is presented in both intervals, only one of which contains the task relevant-feature. It follows that above-chance discrimination in conditions in which participants judge the feature to be as visible in feature-present and feature-absent intervals indicates unconscious perception. Above-chance discrimination indicates that participants processed the task-relevant feature, while their visibility judgments indicate that, to them, seeing the feature felt no different from not seeing it. In an online study, they leveraged this logic to test whether the emotion (i.e., the task-relevant feature) of face stimuli could be perceived unconsciously. In line with previous negative results from 2IFC-based paradigms (Peters, Fesi, et al., 2017; Peters & Lau, 2015), they found no evidence of unconscious perception. More recently, a preprint by Elosegi et al. (2023) applied the same reasoning to the perception of the dominant image category (living vs. nonliving objects) in a stream of images. Strikingly, they showed that healthy volunteers are capable of unconscious ensemble perception. This was the first time that collecting subjective reports via a 2IFC-based task produced evidence of perception without awareness.

**Figure 1.**
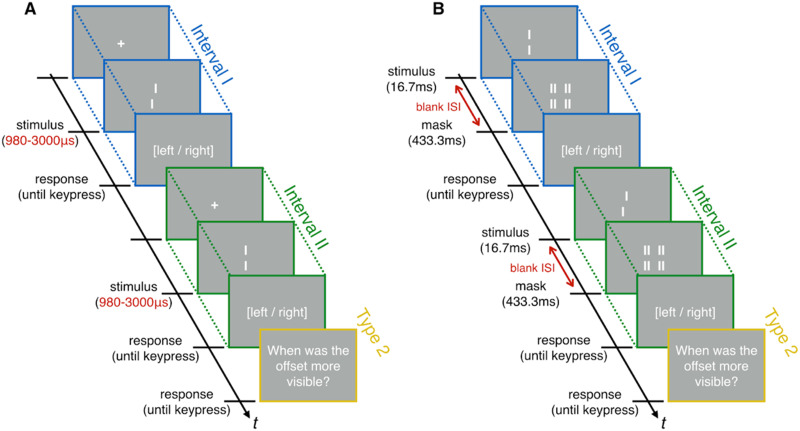
**Stimuli and 2-Interval Forced-Choice procedure for the unmasked (A) and masked (B) conditions.** Targets consisted of two vertical lines displayed on a grey background, either with a horizontal offset to the left/right (offset-present interval) or without an offset (offset-absent interval). In the unmasked condition (A), stimuli were presented between 980 *μ*s and 3000 *μ*s. In the masked condition (B), visibility was determined by the inter-stimulus interval (ISI) ranging from 16.7 ms to 100 ms. After each interval, participants reported the direction of the offset. Then, they indicated in which interval the offset was more visible.

Rajananda et al. (2020) and Elosegi et al. (2023) focused on rather complex stimuli (faces and image streams, respectively). One issue with complex stimuli is that they often contain multiple task-relevant features. It might therefore be more difficult to make sure that participants are not conscious of any task-relevant features, especially given that procedures used to render stimuli unconscious often mask some features, but not others (e.g., Kim & Chong, 2021; Koivisto & Neuvonen, 2020). Here, we use a similar approach to test whether simple visual stimuli can be discriminated without awareness of the task-relevant feature. Vernier stimuli seem to be good candidates for the kind of stimuli that could be unconsciously perceived. First, they can be efficiently masked using metacontrast masking (Herzog et al., 2014). Second, there is evidence that Verniers are still processed by the visual system even when they are masked, as indicated by long-lasting postdictive effects (Drissi-Daoudi et al., 2019; Scharnowski et al., 2007; see Michel and Doerig (2022) for an analysis of what these effects indicate for theories of consciousness). In our experiment, participants discriminated the direction of the horizontal offset of a Vernier stimulus in two intervals. We presented a stimulus in both intervals, but only one was informative with respect to the discrimination task. Each trial thus consisted of an offset-present interval (OP, i.e., a Vernier stimulus with either a left or right offset), and an offset-absent interval (OA, i.e., a neutral Vernier in which the two line segments are vertically aligned) (Figure 1). In each trial, observers indicated in which interval the Vernier offset was more visible as well as the direction of the offset in each interval (even when no offset was present). We manipulated the visibility of the Vernier stimuli in a block-by-block design, either by changing the inter-stimulus interval (ISI) between the Vernier and a mask (Figure 1B), or by presenting unmasked Verniers for durations ranging from 980 to 3000 micro-seconds (Figure 1A) using a modern-day Tachistoscope (Beauny et al., 2020). Backward masking is a popular technique for reducing stimulus visibility without impoverishing the stimulus and guidelines for effectively masking Vernier stimuli are available (Duangudom et al., 2007). However, masking has been criticized on several grounds (Balsdon & Clifford, 2018; Eriksen, 1980). There are reported cases of masking inducing a perception of motion (Ansorge et al., 2009) or influencing the perceived location of the stimulus (Sigman et al., 2008). Further, the stimulus-mask interaction might change at different ISIs, such that two sequential percepts are produced at longer ISIs, while one integrated percept is induced at short ISIs (Jannati & Di Lollo, 2012). For these reasons, we decided to also capitalize on the microsecond-level temporal precision of the Tachistoscope to produce subjectively invisible unmasked stimuli. Using the two techniques in parallel, we can assess the impact of either methodology on our conclusions.

To further explore unconscious vision in our participants, we compared their behavior to the predictions of a set of ideal Bayesian observers, inspired by the work of Peters and Lau (2015). Our strategy is to develop a model-based assessment of unconscious perception (Michel, 2023). We use ideal Bayesian observers to simulate what performance would have been like, had the subjects consciously experienced the task-relevant features. We then use this as our benchmark to identify unconscious perception. Our main goal was to assess the extent to which participants had access to sensory evidence when expressing visibility judgments. Thus, we simulated and fitted seven observer models, all based on Bayesian inference and 2-dimensional Signal Detection Theory (King & Dehaene, 2014; Macmillan & Creelman, 2004). Testing multiple models allowed us to inspect whether our results would generalize to different response strategies. While previous work using these models in the context of 2IFC-based tasks has focused on mimicking the production of confidence judgements (Peters, Fesi et al., 2017; Peters & Lau, 2015; Rajananda et al., 2020), we introduce a set of models explicitly designed to simulate visibility judgements, hoping that this could foster a better understanding of the mechanisms behind perceptual awareness.

## METHODS

### Participants

Twelve participants (age: 18–28; 8 female; all right-handed) were recruited through social media and gave informed consent to participate in our experiment. All volunteers had normal or corrected to normal sight. The experiment was approved by the ethics committee at Université libre de Bruxelles (Comité d’Avis Éthique de la Faculté des Sciences Psychologiques et de l’Éducation). Participants took part in 3 experimental sessions lasting 2 hours each and were paid 18€ per session.

### Stimuli and Apparatus

Targets were Vernier stimuli. They consisted of two 0.07° × 1.15° silver lines displayed at the center of the screen (one line above the horizontal midline, the other below) on a gray background. In the offset-present (OP) interval, line centers were separated 1.26° vertically and ±0.05° horizontally. In the offset-absent (OA) interval, line centers were 1.26° apart vertically and 0° apart horizontally. In the masking condition, stimuli were presented for 16.7 ms. Following Duangudom et al. (2007), masking was achieved by presenting 8 superimposed silver lines for 433 ms which were shifted ±0.23° and ±0.46° horizontally from the centered stimuli, with six levels of inter-stimulus intervals ranging from 16.7 to 100ms (linearly spaced). During the inter-stimulus intervals, only the gray background was displayed. In the unmasked condition, stimuli were presented at eight different durations, between 980 *μ*s and 3000 *μ*s (logarithmically spaced). These presentation speeds were achieved by presenting stimuli on a modern-day LCD-based Tachistoscope (based on Sperdin et al. (2013); see the Appendix of Beauny et al. (2020) for details). Briefly, the Tachistoscope is composed of two LCD screens reflecting on a semi-transparent mirror. The rapid switching on and off of the screens allows to control the duration of visual stimuli with a precision of 1 *μ*s (this precision holds for durations up to 16ms). PsychoPy (v.3.2.4, Peirce et al., 2019) was used to present the stimuli and record participants’ responses.

### Procedure

Volunteers were seated, looking into the Tachistoscope at a viewing distance of 40 cm from the screen. Following Peters and Lau (2015), our procedure consisted of two intervals in each trial, both containing a Vernier stimulus. Only one interval (OP) contained the target (a left/right offset). Participants were required to indicate the orientation of the offset on both OP and OA intervals. Following both intervals, the message “In which interval was the offset more visible” was displayed, and participants pressed a key to indicate whether the offset was more visible in the first or second interval. No feedback was provided.

At the beginning of each session, participants were reminded to try to be as accurate as possible for both their discrimination and visibility judgments. Participants alternated between masked and unmasked blocks in a counterbalanced, randomized design. Each volunteer performed seventeen blocks across three sessions, for a total of about 1900 trials per participant, spread across the two conditions, ISIs and presentation speeds. On average, this resulted in 137 trials per ISI in the masking condition (5 participants performed 128 trials per ISI, 7 performed 144 trials per ISI), and 135 trials per duration level in the unmasked condition (7 participants performed 128 trials per duration, 5 performed 144 trials per duration). In the masking condition, the Tachistoscope behaved just like an ordinary 60 Hz screen.

### Statistical Analyses

For each participant, for each difficulty level and for each condition, we collapsed data across offset orientation, interval presentation order, and session. To report and visualize all behavioral measures mentioned below, we calculated mean performance across participants at each ISI or presentation speed, as well as standard error of the mean.

All statistical analyses were performed using R (R Core Team, 2023) and RStudio (Posit Team, 2023). Group-level response biases were tested with independent-samples two-tailed t-tests. In all other analyses (see below), we fitted Bayesian mixed-effects logistic models with participants as random effects to the behavioral data, using the *rethinking* package (McElreath, 2020), which is based on RStan (Stan Development Team, 2023). All models below were simulated using a No-U-Turn Sampler (Hoffman & Gelman, 2011) within a Hamiltonian Monte Carlo algorithm with 4 chains and 10^5^ samples per chain (half of them were warm-up samples). Maximum tree-depth was 10 and target acceptance rate was 0.95. All models listed below had well-mixed chains and their parameters were fitted with $\hat{R}$ < 1.01 and sufficient number of effective samples.

Participants were excluded when they did not show an increase in orientation discrimination performance and in OP-interval detection performance with easier visibility conditions (i.e., longer ISIs/durations). This was checked by fitting a logistic regression to individual data, separately for the two tasks. For each participant, we computed a Bayes Factor (BF_10_) where H_1_ hypothesizes a positive slope (half-Gaussian prior: mean = 0, sd = 2.30) and H_0_ hypothesizes a null slope (point prior at 0). Participants with a non-positive slope (i.e., BF_10_ ≤ 1/3) were excluded from the dataset. This was done independently for the masking and Tachistoscope experiments. Since a non-positive slope in either task could signify that the participant did not understand how to perform the task, we reproduced our results by excluding from both experiments those that were already excluded from one of the two (results are reported in Appendix B).

#### Performance Without Awareness.

We fit separate models for orientation discrimination and visibility judgements, but the two models were identical in their specification, which follows here:

$${\text{Correct}}_{i}∼\text{Binomial}\left(1,p_{i}\right)$$

$$\text{logit}\left(p_{i}\right)=a_{\text{participant}}+b_{\text{participant}}{\text{level}}_{i}$$

$$a_{\text{participant}}∼\text{Normal}\left(\bar{a},\sigma_{a}\right)$$

$$b_{\text{participant}}∼\text{Normal}\left(\bar{b},\sigma_{b}\right)$$

$$\bar{a}∼\text{Normal}\left(0,0.25\right)$$

$$\bar{b}∼\text{Normal}\left(0,1\right)$$

$$\sigma_{a}∼\text{Exponential}\left(1\right)$$

$$\sigma_{b}∼\text{Exponential}\left(1\right)$$

The correctness of the response (either the orientation discrimination or the visibility judgement) for a stimulus of visibility level *i* was modelled as a binomial process with success probability *p*_*i*_. The latter is a logistic regression of stimulus difficulty. The threshold *a* and slope *b* of the regression was determined for each participant; $\bar{a}$ and $\bar{b}$ are the population-level threshold and slope.

To set better informed priors, visibility levels (i.e., ISIs and presentation durations) were divided by the highest level, making them fit in a 0 to 1 range. The $\bar{a}$ prior expects the group-level threshold to lay in the range 0.38 < *p* < 0.62 with 95% probability. The $\bar{b}$ prior loosely allows both positive and negative effects of visibility level. Weak priors were set for the population-level variance of *a* and *b*. Population-level posterior predictions for each difficulty level were extracted from the fitted models. Independently for orientation discrimination and visibility judgment, we calculated the Bayes Factor between two hypotheses: H_0_ predicted performance to be at chance, H_1_ predicted it being better than chance. Priors for H_0_ and H_1_ were set following Dienes (2019). H_0_ had a Normal prior centered at 0.5 performance with standard deviation of 0.005. H_1_ had a half-Normal prior (upper tail), centered at 0.5 performance. For each difficulty level *i*, the maximum performance one could expect is that at the easier difficulty level *i* + 1. Thus, the standard deviation of the H_1_ prior was set to half of the difference between predicted performance at difficulty *i* + 1 and chance performance. For the easiest difficulty level, the standard deviation was set to 0.25.

In addition to fitting the models to the entire datasets, we fitted them separately to trials in which the task-relevant feature was in the first vs. in the second interval. This enabled testing for the effect of attribute amnesia (Fu et al., 2023). After fitting, we extracted population-level posterior predictions about performance differences depending on which interval contained the offset. Independently for each difficulty level, we calculated the Bayes Factor between two hypotheses. H_0_ predicted equal performance (point prior centered at 0). H_1_ predicted a performance difference (two-tailed Gaussian prior, mean = 0, sd = 0.1).

#### Metacognitive Sensitivity.

We fit a separate model for Type-2 Hit and False Alarm Rates. Type-2 Hits are trials in which the orientation discrimination was correct and the OP interval was indicated, while a Type-2 False Alarm occurs when the orientation is incorrectly reported, but the OP interval is indicated (Fleming & Lau, 2014; Maniscalco & Lau, 2012). The model was defined exactly like in the previous paragraph. Correct orientation discrimination trials were used to fit the model of Type-2 Hits and the incorrect ones were used for the Type-2 False Alarm model.

After fitting, we produced the predicted population-level between Type-2 Hit rates and False Alarm rates. For each difficulty level, we calculated the Bayes Factors between two hypotheses, one predicting the difference to be null (H_0_) and one predicting it to be positive (H_1_). Priors for H_0_ and H_1_ were set following the rationale detailed in the previous section. Two differences should be noted: (1) priors were centered at 0 and (2) the standard deviation of the H_1_ prior was set to half of the predicted HR-FAR difference at difficulty *i* + 1. For the easiest difficulty level, the standard deviation of H_1_ was set to 0.5.

#### Conditional Orientation Discrimination.

We analyzed orientation discrimination performance conditional on having indicated (or not) the OP interval. For each of the two kinds of trials one model was fitted, using again the same specification detailed above. One model was fitted with trials in which OP was chosen, the other with trials in which OA was chosen. We tested, for each difficulty level, whether there would be differences in the predicted population-level discrimination performances. Bayes Factors (between H_0_ predicting no difference and H_1_ predicting performance to be better when OP was chosen) were calculated as explained in the previous paragraph.

#### Response Biases.

Group-level response biases were analyzed separately for the orientation discrimination and interval selection tasks. First of all, they were tested with independent-samples two-tailed t-tests. Then, for each task, we fit the following mixed-effects threshold model, in which the probability of producing a response is modeled as a binomial process (we modeled the probability of reporting a left-ward offset in the orientation discrimination task and the probability of reporting the first interval during the interval selection task):

$$\text{Response}∼\text{Binomial}\left(1,p\right)$$

$$\text{logit}\left(p\right)=a_{\text{participant}}$$

$$a_{\text{participant}}∼\text{Normal}\left(\bar{a},\sigma_{a}\right)$$

$$b_{\text{participant}}∼\text{Normal}\left(\bar{b},\sigma_{b}\right)$$

$$\bar{a}∼\text{Normal}\left(0,1\right)$$

$$\sigma_{a}∼\text{Exponential}\left(1\right)$$

For each model, we calculated the Bayes Factors between two hypotheses. H_0_ predicted the group-level probability of reporting a leftward offset (or the first interval) to be equal to chance (point prior centered at 0.5). H_1_ predicted a small deviation from chance-level (two-tailed Gaussian prior with mean = 0.5 and sd = 0.025, designed to test for small deviations from chance performance).

### Ideal Observer Model

We modeled seven Bayesian observers based on 2-dimensional signal detection theory (2D-SDT) (Macmillan & Creelman, 2004). The starting point was the main model from Peters and Lau (2015), together with its hierarchical counterpart (see Appendix 5 – Alternative model 1 from Peters and Lau (2015)). Following King and Dehaene (2014), all models are based on a Cartesian space, where the two main axes represent evidence in favor of a leftward offset and evidence in favor of a rightward offset. All models were simulated and fitted using MatLab (v. 2021b, The MathWorks Inc., 2021a) and the Parallel Computing Toolbox (v. 7.5, The MathWorks Inc., 2021b). Here follows the general functioning of all models; below we describe each model in greater detail.

During each trial, the observer draws two evidence samples, one for the offset-present interval (*d*_*OP*_) and one for the offset-absent interval (*d*_*OA*_). Evidence sources are modeled as bivariate Gaussian distributions of the form *N*(*μ*, Σ), such that evidence for each stimulus orientation occupies its own dimension. The distribution’s mean for a stimulus of evidence strength *c* is represented as the point [*c*, 0] for the left orientation or as [0, *c*] for the right orientation, whereas the variance-covariance matrix is Σ = [1 0; 0 1]. Thus, evidence samples come in the form *d* = [*d*_*left*_, *d*_*right*_]. For each interval, the model calculates the posterior probability that the sample was produced by a stimulus with leftward offset and, separately, by a stimulus with a rightward offset. The orientation discrimination choice is achieved by determining the highest of the two posteriors. Finally, to determine the offset-present interval, the various models use different kinds of heuristics, some designed to mimic confidence judgements, others representing visibility judgements.

**Figure 2.**
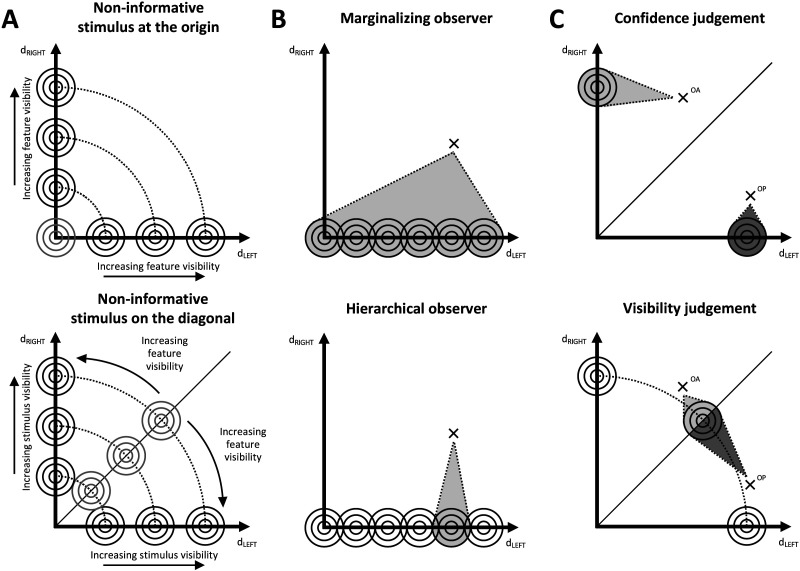
**Attributes of the Bayesian observer models.** Our seven observers result from the various combinations of these attributes. Each model’s evidence space is set in a Cartesian space where the main axes represent evidence in favor of a leftward (d_LEFT_) or rightward offset (d_RIGHT_). Stimuli of different intensities are generated from bivariate Gaussian distributions (represented as concentric circles). Crosses represent bidimensional evidence samples. **(A)** We implemented two different structures of the evidence space. Some models (A-upper) represent the non-informative stimulus (in gray) at the origin of axes. Stimuli with more visible task-relevant features are represented further away from the origin. Other models (A-lower) represent the absence of a stimulus at the origin, while non-informative stimuli are produced by sources on the diagonal (in gray). Here, distance from the origin represents stimulus visibility, while distance from the diagonal represents visibility of the task-relevant feature. **(B)** We also implemented two different ways of accounting for the strength of the stimulus. Some models (B-upper) perform the orientation discrimination and interval selection on a set of potential signal sources at various levels of stimulus strengths, over which they then marginalize. Other observers (B-lower) proceed in a hierarchical fashion. First, they compute the most likely evidence strength of the stimulus (the highlighted signal source), then they perform the two main tasks. **(C)** Finally, we modeled the interval selection process in two ways. To aid visualization, we illustrate here the case of a hierarchical observer with the non-informative signal source placed on the diagonal. Some models (C-upper) judge the interval where the task-relevant feature was most visible by comparing their confidence in the orientation discrimination response across the two intervals. For each interval (OP and OA), our example model determined one most likely signal source. Darker shades mean higher confidence in the discrimination response. Otherwise, some models (C-lower) mimic a visibility judgment by choosing the interval in which the stimulus was the least likely to have been produced by a non-informative signal source (darker shade represents higher probability that the sample actually contained a task-relevant feature). OA = offset-absent interval. OP = offset-present interval.

Here we briefly summarize the three categorical aspects in which our seven models differ from one another:

1. The signal sources for the OA Vernier can be placed in the evidence space in two ways. Some models (1, 2, 5) locate them at the origin of the Cartesian axes (as in Figure 2A-upper). Other models (3, 4, 6, 7) place the neutral Vernier sources on the main diagonal, while the origin represents the absence of a stimulus (Figure 2A-lower).
2. Some observers make explicit use of information about the stimulus evidence strength, while others do not. The former (models 2, 4, 7) produce an estimate of the evidence strength, which is then used to hierarchically perform the orientation discrimination and interval selection tasks (Figure 2B-lower). The latter models (1, 3, 6) directly perform these tasks by marginalizing across possible stimulus strengths (Figure 2B-upper).
3. The interval selection response can be simulated either as a confidence-based judgment (models 1, 2, 3, 4; Figure 2C-upper) or as a visibility-based judgment (models 5, 6, 7; Figure 2C-lower).

The seven models described below are the combinations of these model attributes (see Tables 1–2; the hierarchical and marginalizing visibility models with the neutral Vernier at the origin are equivalent, therefore seven and not eight models were generated).

**Table 1.** Attributes combinations and cross-validated log-likelihood scores (CVlogL) for each Bayesian observer model, relative to the task with masked stimuli.

Model	Masked stimuli						
	Model attributes			Full dataset		Incorrect orientation discrimination trials	
	Non-informative signal source position	Hierarchical or marginalizing	Type of judgment	Ideal	Noisy	Ideal	Noisy
1	Origin	Marginalizing	Confidence	**−930 ± 48**	**−930 ± 49**	−397 ± 34	**−396 ± 34**
2	Origin	Hierarchical	Confidence	−947 ± 41	**−928 ± 48**	−402 ± 33	**−400 ± 33**
3	Diagonal	Marginalizing	Confidence	−935 ± 44	**−929 ± 48**	**−397 ± 34**	**−397 ± 33**
4	Diagonal	Hierarchical	Confidence	−931 ± 48	**−930 ± 49**	−400 ± 34	**−399 ± 34**
5	Origin	Non applicable	Visibility	**−947 ± 48**	**−947 ± 48**	**−397 ± 33**	**−397 ± 33**
6	Diagonal	Marginalizing	Visibility	−935 ± 48	**−934 ± 48**	−400 ± 33	**−397 ± 33**
7	Diagonal	Hierarchical	Visibility	−929 ± 48	**−928 ± 48**	−399 ± 34	**−398 ± 34**

*Note*. The evidence space can be modeled to have the non-informative signal source (representing the neutral Vernier) either at the origin of the evidence axes or on the diagonal (while the origin represents the absence of a stimulus). For some models, the evidence strength of the stimulus is a nuisance variable, and they marginalize across a set of potential evidence strengths; conversely, other models proceed in a hierarchical fashion, by first computing the most likely evidence strength of the stimulus and then using that information to perform the orientation discrimination and interval selection. Finally, some models select the interval in which they were more confident in the orientation discrimination, while others select the interval in which the task-relevant feature was most visible. See Figure 2 and Methods section for further detail. CVlogL scores are reported as cross-subject means alongside the standard error of the mean. For each model we report the CVlogL for the ideal observer (*σ*_*D*_ = 0) and the CVlogL for the noisy observer (*σ*_*D*_ as free parameter). Both measures were calculated once by fitting the full dataset and once by fitting only to incorrect orientation discrimination trials. The highest score between the ideal and noisy version of each model is displayed in **bold**.

**Table 2.** Attributes combinations and cross-validated log-likelihood scores (CVlogL) for each Bayesian observer model, relative to the task with unmasked stimuli.

Model	Unmasked stimuli						
	Model attributes			Full dataset		Incorrect orientation discrimination trials	
	Non-informative signal source position	Hierarchical or marginalizing	Type of judgment	Ideal	Noisy	Ideal	Noisy
1	Origin	Marginalizing	Confidence	**−1290 ± 41**	**−1290 ± 41**	**−565 ± 25**	**−565 ± 25**
2	Origin	Hierarchical	Confidence	−1323 ± 39	**−1296 ± 42**	−573 ± 24	**−568 ± 25**
3	Diagonal	Marginalizing	Confidence	**−1290 ± 41**	**−1290 ± 41**	**−565 ± 25**	**−565 ± 25**
4	Diagonal	Hierarchical	Confidence	**−1297 ± 41**	**−1297 ± 41**	**−564 ± 25**	**−564 ± 25**
5	Origin	Non applicable	Visibility	−1297 ± 40	**−1294 ± 41**	**−569 ± 25**	**−569 ± 25**
6	Diagonal	Marginalizing	Visibility	−1300 ± 41	**−1299 ± 42**	−576 ± 25	**−573 ± 25**
7	Diagonal	Hierarchical	Visibility	**−1290 ± 41**	**−1290 ± 41**	**−563 ± 25**	**−563 ± 24**

*Note*. See the caption of Table 1 for information about model attributes (and Figure 2 and the Methods section for further detail). CVlogL scores are reported as cross-subject means alongside the standard error of the mean. For each model we report the CVlogL for the ideal observer (*σ*_*D*_ = 0) and the CVlogL for the noisy observer (*σ*_*D*_ as free parameter). Both measures were calculated once by fitting the full dataset and once by fitting only to incorrect orientation discrimination trials. The highest score between the ideal and noisy version of each model is displayed in **bold**.

#### 1. Marginalizing Confidence Model (Non-Informative Source at the Origin).

This corresponds to the main model presented by Peters and Lau (2015). Here, we simulated a signal source at the origin to represent neutral Vernier stimuli that have no offset (as in Figure 2A-upper). During each interval, a sample is drawn and the observer calculates the joint probability of evidence strength *c* and orientation *S* using Bayes’ rule:

$$p\left(S,c|d\right)=\frac{p\left(d|S,c\right)p\left(S,c\right)}{p\left(d\right)}$$

Then, the observer marginalizes across evidence strength to estimate the posterior probability of each orientation (as in Figure 2B-upper):

$$p\left(S|d\right)=\int p\left(S,c|d\right)dc$$

The two orientations are assigned the same prior probability of 0.5. The absence of a stimulus orientation has a null prior, as observers are not informed about the possibility of this event. Then the model performs the orientation discrimination, by choosing the most probable *S*:

$$S_{\text{chosen}}=\underset{i}{\text{argmax}}p\left(S_{i}|d\right)$$

Finally, the observer uses the posterior probability associated to *S*_*chosen*_ as an indicator of its confidence in the correctness of the orientation discrimination. The interval with the highest confidence is then chosen (Figure 2C-upper). To do so, the posteriors from the offset-present (OP) and offset-absent (OA) interval are compared by calculating a decision variable *D*:

$$D=\log\left(\frac{p\left(S_{\text{chosen},OP}|d_{OP}\right)}{p\left(S_{\text{chosen},OA}|d_{OA}\right)}\right)$$

When *D* exceeds 0, the offset-present interval is chosen. Otherwise, the offset-absent interval is selected.

#### 2. Hierarchical Confidence Model (Non-Informative Source at the Origin).

This model is a variant of model 1 and was previously tested by Peters and Lau (2015) as a “hierarchical observer”. The previous observer integrated across evidence strength *c*, while here this is treated as a nuisance variable. Thus, this model first estimates the most likely evidence strength of the stimulus for both orientations *i*:

$${ĉ}_{i}=\underset{c_{i}}{\text{argmax}}p\left(d|S_{c_{i}}\right)$$

Then, the posterior probabilities for the most likely evidence strength are calculated (as in Figure 2B-lower):

$$p\left(S_{{ĉ}_{i}}|d\right)=\frac{p\left(d|S_{{ĉ}_{i}}\right)p\left(S_{{ĉ}_{i}}\right)}{p\left(d\right)}$$

All subsequent steps are identical to model 1, just like all parameters that were not mentioned in this subsection.

#### 3. Marginalizing Confidence Model (Non-Informative Source on the Diagonal).

This model is designed exactly like model 1, with one key difference. The origin of the evidence space represents the absence of a stimulus. The source of a non-informative stimulus of strength *c* is centered at point [*c*/$\sqrt{2}$, *c*/$\sqrt{2}$], meaning at *c* distance from the origin, in the middle of the arch connecting informative sources [*c*, 0] and [0, *c*] (as in Figure 2A-lower). Thus, during each trial, the samples from the offset-present and offset-absent intervals are drawn from sources centered at the same distance *c* form the origin. This configuration seemed more appropriate for experiments like ours, in which the non-informative interval is not empty, but contains a neutral stimulus. The advantage of such a configuration is that it allows to account, within the same model, for the visibility of both the stimulus and its task-relevant feature (i.e., the offset). The former is represented by the distance of the signal source from the origin, the latter by the distance between the source of an oriented signal and the source of a neutral signal. This configuration intrinsically accommodates the fact that more salient stimuli (i.e., longer, more contrasted, etc.) are also easier to discriminate, since the sources for the left-oriented and right-oriented stimuli are more distant. Moreover, by varying their distance from the origin, it is possible to represent non-informative stimuli of different strength, which is not possible when using the former configuration.

#### 4. Hierarchical Confidence Model (Non-Informative Source on the Diagonal).

This model is designed exactly like model 2, with the only difference that its evidence space is constructed like in model 3. Thus, here the origin represents the absence of a stimulus, whereas the source of a non-informative stimulus of strength *c* is centered at point [*c*/$\sqrt{2}$, *c*/$\sqrt{2}$]. During each trial, the samples from the offset-present and offset-absent intervals are drawn from sources centered at the same distance *c* form the origin (Figure 2A-lower).

#### 5. Visibility Model (Non-Informative Source at the Origin).

This model is a variant of model 1, designed to mimic the process of selecting the interval based on a visibility judgment (relative to the task-relevant feature of the stimulus). The only difference with model 1 is the way the decision variable *D* is computed. For each interval *j*, the observer evaluates the probability that the stimulus contained an offset and then computes *D* based on these probabilities (see Figure 2C-lower). To achieve that, it first calculates the likelihood *p*(*d*|*N*_*j*_) that the evidence sample was produced by the non-informative signal source *N* placed at the origin. Then, the likelihood is used to calculate the posterior probability that a stimulus with no offset had been shown in either interval *j*:

$$p\left(N_{j}|d_{j}\right)=\frac{p\left(d_{j}|N_{j}\right)p\left(N_{j}\right)}{p\left(d_{j}\right)}$$

where the prior *p*(*N*_*j*_) is 0.5 for both intervals. Finally, the probability of an interval containing a stimulus with an offset is simply computed as 1 − *p*(*N*_*j*_|*d*_*j*_) and the posteriors for the two intervals are then compared to produce the decision variable *D*, as follows:

$$D=\log\left(\frac{1-p\left(N_{OP}|d_{OP}\right)}{1-p\left(N_{OA}|d_{OA}\right)}\right)$$

As with all the previous models, when *D* exceeds 0, the offset-present interval is chosen. Otherwise, the offset-absent interval is selected.

#### 6. Marginalizing Visibility Model (Non-Informative Source at the Diagonal).

This model also mimics the process of producing visibility judgements (with a rationale similar to model 5; see Figure 2C-lower) but using the evidence space configuration of model 3 (Figure 2A-lower). This means that, here, the origin of the axes represents the absence of a stimulus and that a non-informative stimulus of strength *c* is generated from a bivariate distribution centered at [*c*/$\sqrt{2}$, *c*/$\sqrt{2}$]. Another feature in common with model 3 (and 1) is the marginalization over possible levels of evidence strength (Figure 2B-upper). Thus, the process of discriminating the offset orientation is carried out exactly as in model 3. For clarity, the computations are the exact same as in model 1, just applied to a different configuration of signal sources. To produce the visibility judgement, observer F calculates, separately for each interval *j*, the posterior probability that the stimulus was generated by a non-informative source *N* with evidence strength *c*:

$$p\left(N_{j},c|d_{j}\right)=\frac{p\left(d_{j}|N_{j},c\right)p\left(N_{j},c\right)}{p\left(d_{j}\right)}$$

The prior *p*(*N*_*j*_, *c*) is set to 0.5 for both intervals. Then, the posteriors are marginalized across all levels of *c*, as follows:

$$p\left(N_{j}|d_{j}\right)=\int p\left(N_{j},c|d_{j}\right)dc$$

Following the rationale from model 5, the marginalized posteriors are then subtracted from 1 and used to compute a decision variable *D*:

$$D=\left(\frac{1-p\left(N_{OP}|d_{OP}\right)}{1-p\left(N_{OA}|d_{OA}\right)}\right)$$

#### 7. Hierarchical Visibility Model (Non-Informative Source on the Diagonal).

The last model is the hierarchical variant of model 6. The two models share the same evidence space setting (Figure 2A-lower) and the same rationale for producing visibility judgements (Figure 2C-lower). However, this model computes the most likely evidence strength of the stimulus $\hat{c}$ as an intermediate variable (as in Figure 2B-lower), which is then used to compute the orientation discrimination and the interval decision. For each orientation *i*, the model computes:

$${ĉ}_{i}=\underset{c_{i}}{\text{argmax}}p\left(d|S_{c_{i}}\right)$$

Then, it chooses the orientation with the highest posterior relative to the most likely evidence strength:

$$p\left(S_{{ĉ}_{i}}|d\right)=\frac{p\left(d|S_{{ĉ}_{i}}\right)p\left(S_{{ĉ}_{i}}\right)}{p\left(d\right)}$$

$$S_{\text{chosen}}=\underset{i}{\text{argmax}}p\left(S_{{ĉ}_{i}}|d\right)$$

This observer uses the intermediate variable $\hat{c}$ to compute the probability that the samples contained an offset. The rationale is the same as in model 6, but here applied only to the most likely evidence strength for the chosen orientation $\hat{c}$_*chosen*_. First, the posterior probability that the stimulus was generated by the non-informative source *N* with evidence strength $\hat{c}$_*chosen*_ is calculated, separately for each interval *j*:

$$p\left(N_{j,{ĉ}_{\text{chosen}}}|d_{j}\right)=\frac{p\left(d_{j}|N_{j,{ĉ}_{\text{chosen}}}\right)p\left(N_{j,{ĉ}_{\text{chosen}}}\right)}{p\left(d_{j}\right)}$$

where the prior *p*($N_{j,{\hat{c}}_{\text{chosen}}}$) is set the 0.5 for each interval. Finally, the marginalized posteriors are subtracted from 1 and used to compute a decision variable *D*, just like in models 5 and 6:

$$D=\left(\frac{1-p\left(N_{OP,{ĉ}_{\text{chosen}}}|d_{OP}\right)}{1-p\left(N_{OA,{ĉ}_{\text{chosen}}}|d_{OA}\right)}\right)$$

#### Manipulating Access to Sensory Evidence.

All the models defined above make full use of the information contained in the evidence samples to judge which interval had the most visible offset. To simulate a corruption of this process, Gaussian noise *δ* ~ *N*(0, *σ*_*D*_) with varying magnitudes *σ*_*D*_, is added to this decision variable *D*:

$$D_{\text{noisy}}=D+\delta$$

A corruption of the ability to discriminate the interval with the task-relevant feature, while the orientation discrimination process remains intact, indicates performance without awareness. Hence, the parameter *σ*_*D*_ can be manipulated to simulate observers capable of unconscious perception in varying degrees. For models 1–4, we simulated all possible *σ*_*D*_ values between 0 and 1 in steps of 0.01. For models 5–7, we simulated *σ*_*D*_ values between 0 and 10 in steps of 0.1. The difference is due to the visibility models being more study to this noise injection.

### Model Fitting and Comparison

Each observer was fit separately for each participant and for data from each of the two tasks. Our models are defined only for orientation discrimination accuracies between 0.5 and 1. Thus, we calculated percent correct orientation discrimination for each difficulty level using a parametric approach (Moscatelli et al. (2012); after collapsing data across offset orientation, interval presentation order, and session).

The next step was to determine, for each participant, a set of evidence strengths that would allow the Bayesian observer to perform as close as possible to the participant in the orientation discrimination task. Therefore, for each model we simulated 10^5^ trials at all evidence strengths *c* between 0 and 5 (in steps of 0.1). Then, we used a genetic algorithm (Conn et al., 1997) to find the set of evidence strengths that maximizes the multinomial likelihood function (Dorfman & Alf, 1968):

$$L\left(\phi |\text{data}\right)=\prod_{ij}P_{\phi }{\left(R_{i}|c_{j}\right)}^{n_{\text{data}}\left(R_{i}|c_{j}\right)}$$

Here, *ϕ* is the set of parameters, meaning the set of evidence strengths to be fitted, one per difficulty condition in the behavioral task (i.e., 6 parameters for the masking task, 8 for the task with unmasked stimuli). For each trial, the available responses *R*_*i*_ are “correct” or “incorrect”. *P*_*ϕ*_(*R*_*i*_|*c*_*j*_) is the probability of correctly discriminating the offset when presented with a stimulus of strength *c*_*j*_, as predicted by a model with evidence strengths *ϕ*. *n*_*data*_(*R*_*i*_|*c*_*j*_) is the count of trials in which the participant was shown a stimulus with strength *c*_*j*_ and provided response *R*_*i*_. The use of a genetic algorithm is motivated by (1) its applicability to discrete functions (as is the case here, where only 51 levels of *c* were simulated) and (2) the possibility of including inequality constraints to parameter values. Like so, data relative to easier conditions was constrained to be fit to a higher *c* than data relative to harder conditions. This is crucial for handling situations in which a participant performs slightly better in a condition that is supposed to be harder. Using this algorithm made sure that the model fitting would respect the basic assumption that higher values of *c* would be assigned to easier experimental conditions.

Next, we determined the goodness of fit of each model to both orientation discrimination and interval choice responses. All models were fitted both as ideal observers (i.e., *σ*_*D*_ fixed at 0) and noisy observers (i.e., *σ*_*D*_ as free parameter). BIC (Bayesian Information Criterion) scores were computed, as a way to take the number of fitted parameters *k* and the total number of data points *n*_*data*_ into account when estimating goodness of fit:

$$BIC=-2\ln\left(L\left(\phi |\text{data}\right)\right)+k\ln\left(\Sigma n_{\text{data}}\right)$$

Here, *L*(*ϕ*|*data*) is the multinomial likelihood function by Dorfman and Alf (1968):

$$L\left(\phi |\text{data}\right)=\prod_{ijk}P_{\phi }{\left(R_{i}^{ori},R_{j}^{int}|c_{k}\right)}^{n_{\text{data}}\left(R_{i}^{ori},R_{j}^{int}|c_{k}\right)}$$

Responses in both tasks $R_{i}^{ori}$and $R_{j}^{\mathrm{int}}$ only vary between the two outcomes “correct” or “false”, allowing for four possible responses in each trial. Each response at a given stimulus difficulty level *c*_*k*_ has a probability *P*_*ϕ*_ determined by the model and occurs *n*_*data*_ times in the experiment. The BIC for the noisy observer is computed using the best-fitting noisy observer, meaning the one that maximizes *L*(*ϕ*|*data*).

In addition to BIC, we assessed model fitting by means of cross-validation, which has the added value of providing insight into a model’s ability to predict novel data. Note that for the ideal observer models, CV-logLH scores are equivalent to the logarithm of *L*(*ϕ*|*data*) scores, since *σ*_*D*_ is clamped at 0. For the noisy observers, the data was randomly split in 10 subsets, balanced in terms of number of trials per condition. After fitting *σ*_*D*_ on 9 data splits using the likelihood function above, the same function is used to compute the fit of the remaining split *i* to the most-likely noisy observer. A cross-validated log-likelihood score CV-logL is computed as:

$$CVlogL=\log\left(\sum_{i}\frac{L\left(\phi |{\text{data}}_{i}\right)}{N}\right)$$

where *N* is the number of splits. To reduce the influence of data-splits composition on the final score, the whole procedure was repeated 50 times, resampling the data-splits at each round. Scores were averaged across resampling runs.

Further, the two fitting scores detailed above, i.e., BIC and CV-logL, were separately recalculated by taking into account only trials in which participants were incorrect about the offset’s orientation. This provided a measure of the extent to which our models produce accurate predictions of Type-2 False-Alarm rates.

Finally, we also fitted our models separately to the two easiest (ISI = 83.3 ms and 100 ms for masked stimuli, duration = 2557 *μ*s and 3000 *μ*s for unmasked stimuli) and the two hardest (ISI = 16.7 ms and 33.3 ms for masked stimuli, duration = 980 *μ*s and 1150 *μ*s for unmasked stimuli) conditions in each task. The easy conditions were selected as those in which all participants were at least 70% accurate in the orientation discrimination task. When fitting data from the Tachistoscope task, one participant was excluded because his performance was below 70%. The same goes for one participant in the masking task. The hard conditions were selected as those in which all participants were below 70% accuracy in the orientation discrimination task. When fitting data from the masking task, one participant was excluded because his performance was above 70%. We used BIC scores to estimate the best-fitting *σ*_*D*_ for each combination of participant, model and task difficulty (easy vs. hard). We did not replicate this analysis using CV-logL scores since the relative fitting process does not allow extracting best-fitting *σ*_*D*_ scores.

## RESULTS

### Behavioral Results

#### Methods and Paradigm.

Twelve volunteers performed the task in a block-by-block design alternating between blocks of masked (Figure 1B) and unmasked stimuli (Figure 1A). In both conditions, participants were presented with two successive intervals in which they had to discriminate the orientation (right or left) of a Vernier offset (i.e., whether the upper bar was to the left or to the right of the lower bar). Crucially, only one interval (the offset-present, OP, interval) contained an offset. Unbeknownst to participants, the other (the offset-absent, OA, interval) contained a neutral Vernier, with no left/right offset: its orientation was impossible to discriminate. Importantly, participants were not informed that only one of the intervals actually contained the task-relevant feature. After each stimulus presentation, participants indicated the orientation of the offset. Then, they reported in which interval the offset felt more visible (OP and OA intervals were pseudo-randomly ordered).

We excluded 2 participants from the masking task and 1 participant from both tasks because their accuracy in selecting the OP interval did not increase with more salient stimuli. This behavior could indicate that participants did not understand how to correctly judge offset visibility. Since it seems implausible that two participants could follow instructions with masked but not with unmasked stimuli, we reanalyzed all data from the unmasked task by excluding all three observers. This led to results similar to those presented below (see Appendix B).

We set one main, and two additional, criteria for identifying unconscious behavior in our data. All criteria were evaluated using Bayesian mixed-effect logistic regressions (participants as random effects) and calculating Bayes Factors (BFs) at every difficulty level. Crucially, priors for BFs were designed to incorporate the prediction that harder conditions lead to smaller effects.

Our main analysis asked whether participants displayed successful discrimination performance in the absence of awareness. As such, we tested if participants could discriminate the orientation of the offset above chance without being able to judge the offset as more visible in the OP interval compared to the OA interval. This behavior would indeed indicate that, for the participants, the offset successfully discriminated in the OP interval was just as subjectively visible as no offset at all—a valid indicator of subjective invisibility.

The two additional criteria imposed more stringent requirements for identifying offset unawareness. First, participants are not unconscious of the offset if their orientation discrimination accuracy is higher when they report the OP interval as more visible (compared to when they report the OA interval). Therefore, we tested for differences in discrimination performance depending on the accuracy of the interval choice. Second, observers are not unaware of the offset if they are more likely to report the OP interval when they are also correct in the orientation discrimination (compared to when they are incorrect). Thus, we tested for differences between Type-2 Hit and False Alarm rates at each duration/ISI.

For clarity, we present the results from masked and unmasked stimuli in turn. Only key results are reported below (Supplementary Tables 1–2 of Appendix A report all mean performances, BFs and relative scaling factors).

#### Metacontrast Masking.

**Figure 3.**
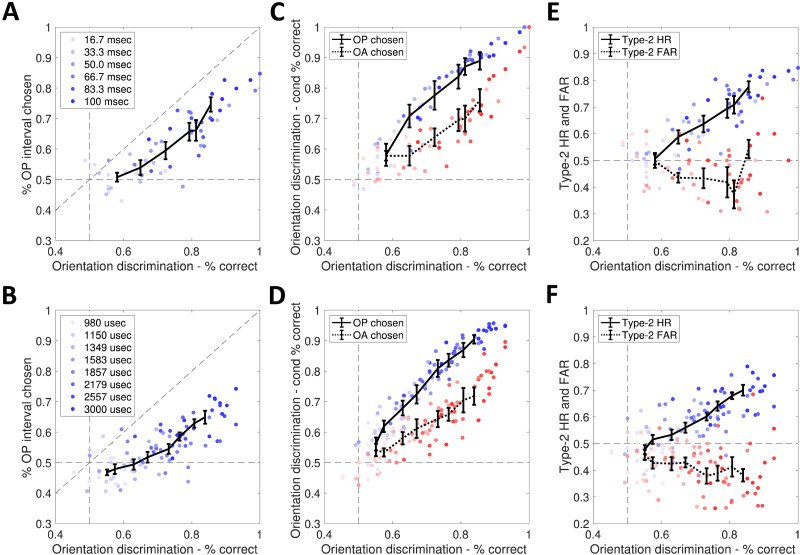
**Behavioral group level results.** Each data point represents performance for a single participant at a given ISI (A, C, E) or presentation speed (B, D, F). Panels A and B represent accuracy in OP (offset-present) interval selection as a function of orientation discrimination performance. Panels C and D show orientation discrimination conditioned on judging the offset as being more visible in the offset-present interval (OP chosen, blue) or not (OA chosen, red). Panels E and F show Type-2 Hits (blue) and False alarm rates (red). All panels represent orientation discrimination accuracy on the horizontal axis. Error bars represent cross-participant mean performances and standard errors. Darker shades of blue/red indicate conditions with longer ISI/duration.

When stimuli were followed by a mask, participants were always able to discriminate the orientation of the offset better than chance, even at the shortest ISI (ISI = 16.7 ms: mean accuracy = 0.580 ± 0.023; BF_10_ = 12.0). For ISIs of at least 33.3 ms, they were also able to identify the offset as being more visible in the OP interval compared to the OA interval (Figure 3A; ISI = 33.3 ms: mean accuracy = 0.539 ± 0.024, BF_10_ = 7.44). However, they failed to perform better than chance at the shortest ISI of 16.7 ms (mean accuracy = 0.508 ± 0.015; BF_10_ = 0.66). Nonetheless, the BF_10_ for the visibility judgment is close to 1, meaning that we have insufficient evidence to conclude that participants were unable to indicate the OP interval better than chance.

Moreover, orientation discriminations were more accurate during trials in which participants indicated the OP interval compared to trials in which they did not (Figure 3C). This was true for all ISIs above 33 ms (ISI = 33.3 ms: mean accuracy given OP chosen = 0.708 ± 0.037, mean accuracy given OA chosen = 0.578 ± 0.032, BF_10_ = 2.84). For the shortest ISI, we could not conclude for or against a difference in performance (ISI = 16.7 ms: mean accuracy given OP chosen = 0.581 ± 0.036, mean accuracy given OA chosen = 0.578 ± 0.016, BF_10_ = 1.24), due to lack of evidence.

Regarding metacognitive performance, participants were much more likely to select the OP interval in trials during which they were also correct about the orientation of the offset (Figure 3E; ISI = 33.3 ms: mean Type-2 Hit rate = 0.588 ± 0.025, mean Type-2 False Alarm rate = 0.435 ± 0.018, BF_10_ = 83.4). We found weak evidence in the same direction, even at ISI = 16.7 ms (mean Type-2 Hit rate = 0.506 ± 0.022, mean Type-2 False Alarm rate = 0.497 ± 0.025, BF_10_ = 2.75).

Additionally, participants were equally likely to identify the OP interval regardless of whether it was presented in the first or in the second interval. This was especially true for the shortest ISI (ISIs = 16.7 ms: mean accuracy (OP 1st) = 0.527 ± 0.028, mean accuracy (OP 2nd) = 0.488 ± 0.038, BF_10_ = 0.44; ISI = 33.3 ms: mean accuracy (OP 1st) = 0.493 ± 0.032, mean accuracy (OP 2nd) = 0.586 ± 0.039, BF_10_ = 0.53), while for the longer ones our evidence was not conclusive (see Supplementary Table 1 for details). On the other hand, orientation discrimination performance was independent of the interval in which the offset was shown at all ISIs (all BF_10_ ≤ 0.51).

Our volunteers showed no preference for reporting leftward or rightward offsets (M = 51.14%, SD = 7.06%, t(8) = 0.485, p = 0.64, BF_10_ = 0.74). However, they had a slight but non-significant bias towards judging the offset as being more visible in the second interval (M = 47.87%, SD = 7.54%, t(8) = −0.850, p = 0.42, BF_10_ = 0.87).

Overall, the use of masks failed to produce strong evidence that discriminating the orientation of Vernier offsets can be performed while remaining unaware of said offsets. We leave open the possibility that the shortest ISI might have induced unconscious perceptual states—our data were not conclusive in this respect.

#### Tachistoscope (Unmasked).

Participants performed the orientation discrimination task above chance at all stimulus durations, including the shortest one (duration = 980 *μ*s: 0.551 ± 0.014, BF_10_ = 6.94; Figure 3B). Interestingly, when stimuli were presented for 980 *μ*s and 1150 *μ*s, participants could not judge the offset to be more visible in the OP interval significantly above chance (duration = 980 *μ*s: 0.468 ± 0.009, BF_10_ = 0.26; duration = 1150 *μ*s: 0.479 ± 0.016, BF_10_ = 0.30). For stimuli of 1349 *μ*s there was insufficient evidence for determining whether they could correctly detect the interval with the offset (0.494 ± 0.017, BF_10_ = 0.91), while we observed significantly above-chance visibility judgments for all longer durations (duration = 1583 *μ*s: 0.517 ± 0.018, BF_10_ = 5.40).

Despite the evidence that Vernier offsets can be discriminated unconsciously (at least at 980 *μ*s and 1150 *μ*s), our additional criteria for unconscious perception were not met (Figure 3D and F). In fact, all conditions showed better discrimination accuracies when the OP interval was selected, compared to when it was not, even though evidence is weak for the 980 *μ*s duration (duration = 980 *μ*s: mean accuracy given OP chosen = 0.561 ± 0.021, mean accuracy given OA chosen = 0.541 ± 0.020, BF_10_ = 2.34; duration = 1150 *μ*s: mean accuracy given OP chosen = 0.619 ± 0.019, mean accuracy given OA chosen = 0.533 ± 0.013, BF_10_ = 4.94).

Additionally, we found a difference in Type-2 Hit and False Alarm rates for all stimulus durations (duration = 980 *μ*s: mean Type-2 Hit rate = 0.477 ± 0.017, mean Type-2 False Alarm rate = 0.458 ± 0.019, BF_10_ = 21.0; duration = 1150 *μ*s: mean Type-2 Hit rate = 0.515 ± 0.017, mean Type-2 False Alarm rate = 0.427 ± 0.021, BF_10_ = 789).

Moreover, orientation discrimination performance was equal regardless of whether the OP interval was displayed first or second (BF_10_ ≤ 0.34 for all stimulus durations). Strikingly, this was not true for interval discrimination performance, where participants were more accurate when the offset was shown in the first interval (duration = 980 *μ*s: mean accuracy (OP 1st) = 0.576 ± 0.026, mean accuracy (OP 2nd) = 0.360 ± 0.025, BF_10_ = 22.4; duration = 1150 *μ*s: mean accuracy (OP 1st) = 0.547 ± 0.018, mean accuracy (OP 2nd) = 0.410 ± 0.031, BF_10_ = 10.6; duration = 1349 *μ*s: mean accuracy (OP 1st) = 0.533 ± 0.025, mean accuracy (OP 2nd) = 0.455 ± 0.036, BF_10_ = 4.29). This effect was absent for stimulus durations equal or longer than 1857 *μ*s, in which we found mild evidence that performance was independent of which interval had the OP stimulus.

In contrast to the masking condition, our volunteers showed a preference towards selecting the “left” answer in the discrimination task (M = 54.34%, SD = 6.16%, t(10) = 2.339, p = 0.04, BF_10_ = 2.27), as well as a (non-significant) bias towards judging the first interval as more visible (M = 52.92%, SD = 6.22%, t(10) = 1.559, p = 0.15, BF_10_ = 1.23).

Overall, we found indications of unconscious discrimination for stimuli presented for 980 *μ*s and 1150 *μ*s, but, when applying stricter criteria, we could not confirm participants’ unawareness.

### Bayesian Ideal Observer Analyses

To better understand the underpinnings of the observed behavior, we modeled ideal observers in a 2-dimensional Signal Detection Theory framework. This approach was previously successful at capturing various features of conscious and unconscious perception (King & Dehaene, 2014), even in the context of 2IFC tasks (Peters, Fesi, et al., 2017; Peters & Lau, 2015; Rajananda et al., 2020).

We simulated and fitted seven observer models, which differed from each other in three categorical aspects:

1. The first is the placement in the evidence space of signal sources for the neutral Vernier. Past models placed the non-discriminable stimulus at the origin of the axes that represent evidence in favor of the two discrimination outcomes (here, left and right). This was done regardless of the fact that the neutral stimulus was a blank screen (Peters, Fesi, et al., 2017; Peters & Lau, 2015) or a non-discriminable element (Rajananda et al., 2020). Some of our observers use this configuration (models 1, 2, 5; Figure 2A-upper), while others represent the absence of a stimulus at the origin and the neutral Vernier sources are placed on the diagonal (3, 4, 6, 7; Figure 2A-lower). The latter are designed to make predictions about both the stimulus and its task-relevant feature within the same evidence space: the source-origin distance represents the visibility of the stimulus, whereas stimuli farther away from the diagonal have higher offset visibility.
2. Observers differ in the way they use information about the stimulus’s evidence strength. Some extract the most likely evidence strength of the stimuli as a nuisance variable and use it for the orientation discrimination and interval selection (models 2, 4, 7; Figure 2B-lower). These observers simulate a “hierarchical” process in which evidence strength estimation is the first-order process upon which other processes depend. In contrast, other models (1, 3, 6) directly perform the two tasks by marginalizing across a vector of potential stimulus strengths (Figure 2B-upper).
3. Thus far, Bayesian observers have been used to simulate a confidence-based interval choice (Peters, Fesi, et al., 2017; Peters & Lau, 2015; Rajananda et al., 2020). In addition to that (models 1, 2, 3, 4; Figure 2C-upper), we simulated a novel strategy where the observer chooses the interval with the highest visibility of the task-relevant feature (5, 6, 7; Figure 2C-lower). To the best of our knowledge, this is the first time that a visibility evaluation is modeled in the context of a 2IFC task.

We generated and compared our models as combinations of these model attributes. Note that two combinations, the hierarchical and marginalizing visibility models that have the neutral Vernier at the origin, are equivalent since they have only one possible source of offset-absent stimuli. Therefore, the combinations of these attributes resulted in seven unique observer models (their attributes are summarized in Tables 1–2).

#### Ideal vs. Noisy Observers.

All seven models include a parameter *σ*_*D*_ that controls the reliability of sensory information available to the observer when making the visibility judgment. By increasing *σ*_*D*_, Gaussian noise of increasing strength is injected in the interval selection process, mimicking the case of a “noisy” observer that is partially unaware of the information used to discriminate the offset direction (*σ*_*D*_ has no influence on the direction discrimination). We fitted each model twice. In one case, *σ*_*D*_ was allowed to vary between 0 and 1 (0 and 10 for visibility models). In the other, *σ*_*D*_ was fixed at 0, simulating an ideal observer with optimal introspective access to sensory evidence.

**Figure 4.**
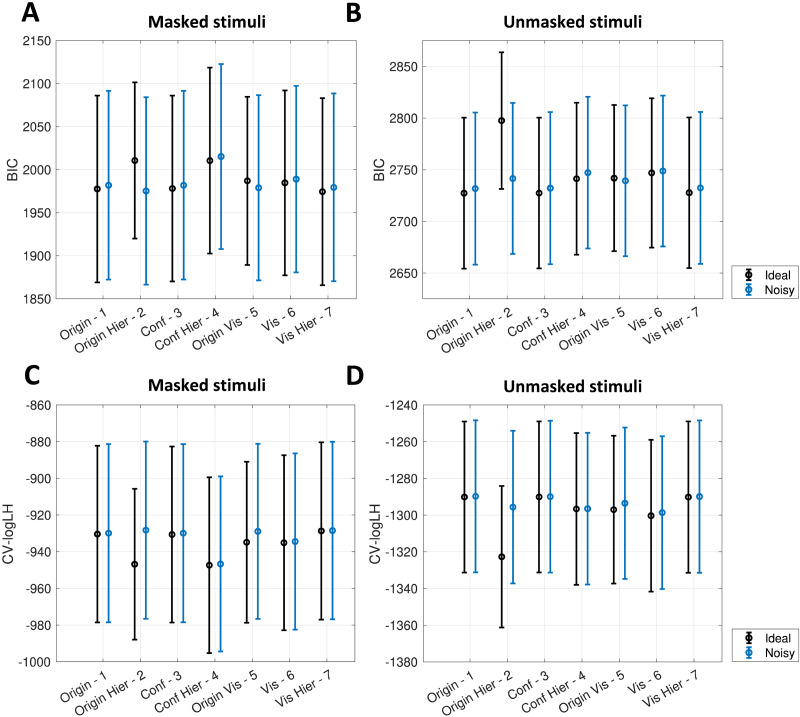
**Ideal vs. noisy Observer models.** For every model, mean BIC (panels A–B) and cross-validated log-likelihoods (CV-logL, panels C–D) are shown. Scores for both ideal (black) and noisy observers (blue) are shown. Error bars represent cross-participant standard errors. Data from the masking experiment (panels A and C) and from the Tachistoscope experiment (panels B and D) were fitted separately.

First, we evaluated model fits using the Bayesian Information Criterion (BIC). Cross-participant mean BIC scores showed better fits for the ideal observer across all models, except two (models 2 and 5). The same was true for masked and unmasked stimuli (Figure 4A and B, respectively; Supplementary Tables 3 and 4 contain all best-fitting *σ*_*D*_ values and mean BIC scores).

BIC scores penalize models with more parameters to prevent overfitting. Since noisy observers have one additional parameter (*σ*_*D*_), we wished to confirm that the best fits of the ideal models reflected an advantage in predicting novel data, in particular because best fitting *σ*_*D*_ values for the noisy models were always quite close to zero (except for model 2). Therefore, we sought to confirm BIC results by fitting our models using 10-fold cross-validation, which allows one to estimate a model’s ability to predict unseen data. Mean log-likelihood (CV-logL) scores indicated a predictive advantage for the “noisy” version of all seven observers (Figure 4C and D, for masked and unmasked stimuli, respectively). However, for some models the difference was so small to be negligible (ΔCV-logL < 1 for models 1, 3, 4 and 7 in both tasks and for model 6 in the masking task). Tables 1 and 2 contain all mean CV-logL scores.

However, our conclusions could be confounded by the presence of sources of noise (other than the impaired access to sensory evidence) influencing the interval selection task. Thus, in order to cross-check our results, we refitted all our models. We did so separately for the two hardest (ISI = 16.7ms and 33.3ms for masked stimuli, duration = 980 *μ*s and 1150 *μ*s for unmasked stimuli) and the two easiest conditions (ISI = 83.3 ms and 100 ms for masked stimuli, duration = 2557 *μ*s and 3000 *μ*s for unmasked stimuli) in each task. Assuming that participants were always aware of sensory information in the easier conditions, the relative best fitting *σ*_*D*_s represent the amount of noise in the 2IFC task that is not due to impaired sensory access. Finding similar best-fitting *σ*_*D*_s for easier and harder conditions would entail that deviations from ideal performance in our dataset do not derive from suboptimal offset awareness. Indeed we found the opposite – our data in the harder conditions is best captured by much higher *σ*_*D*_ levels compared to the easier conditions. This was consistent across all models and both tasks (see Supplementary Table 5 for details).

#### Best Fitting Observers.

**Figure 5.**
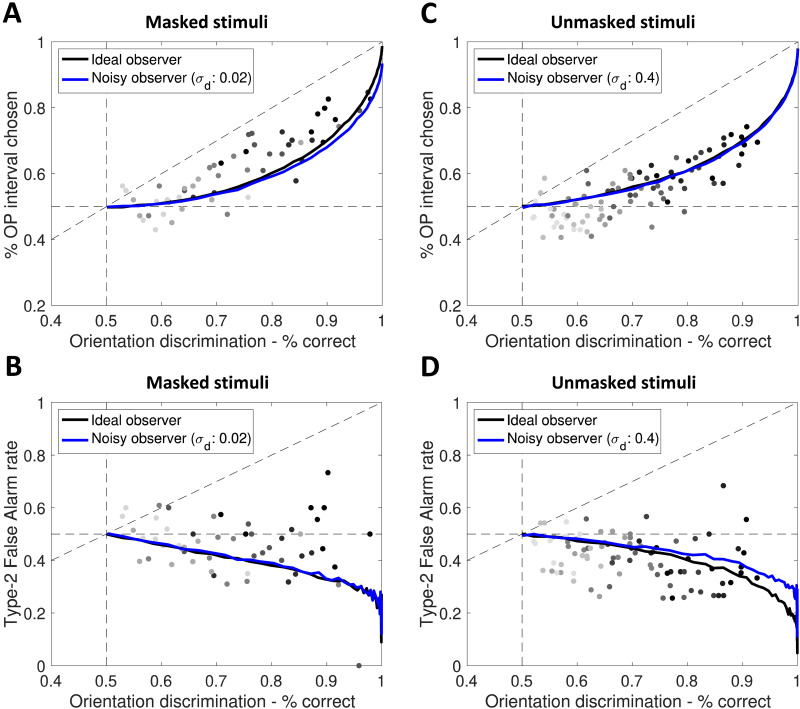
**Best-fitting Bayesian observers.** The marginalizing confidence observer with the evidence space configuration shown in Figure 2A-lower (model 3) was the best at recapitulating data from the masking experiment. Panels A and B show its predictions relative to interval selection and Type-2 False Alarm rates. Behavior in the task with unmasked stimuli was best represented by the hierarchical visibility model (model 7, also simulated in the evidence space of Figure 2A-lower). Its predictions for interval selection (C) and Type-2 False-Alarm rates (D) are shown. Black line: ideal observer (*σ*_*D*_ = 0). Blue line: noisy observer with the closest *σ*_*D*_ to the mean best-fitting *σ*_*D*_. Single dots represent performance for one participant at one ISI/duration (darker dots represent longer ISI/durations).

We tried to establish which of our seven observers (ideal or noisy) would best account for participants’ behavior, using CV-logL scores. Four models best captured data from the masking task (Table 1). They were: the “noisy” version of model 2 (confidence-based hierarchical observer with the neutral Vernier placed at the origin; CV-logL(noisy) = −928 ± 48); model 7 in both versions (hierarchical visibility model with the non-informative signal sources on the diagonal; CV-logL(noisy) = −928 ± 48; CV-logL(ideal) = −929 ± 48), and the noisy version of model 3 (marginalizing confidence observer with the neutral Vernier on the diagonal; CV-logL(noisy) = −929±48). Interestingly, our models make qualitatively different predictions of Type-2 False Alarm rates. Thus, to narrow down this list of models, we fitted CV-logL scores taking into account only incorrect orientation discrimination trials. From the above list, the noisy version of the marginalizing confidence model best accounted for Type-2 False-Alarm rates (model 3; CV-logL(noisy) = −397 ± 33; Figure 5A–B).

When fitting data from the experiment with unmasked stimuli (CVlogL scores are reported in Table 2), three models stood out as best performing, in both the ideal and noisy version. These were the confidence-based marginalizing observer with non-informative Vernier at the origin (model 1; CV-logL(noisy) = −1290 ± 41; CV-logL(ideal) = −1290 ± 41) and two observers with the neutral stimuli on the diagonal: the hierarchical visibility (model 7; CV-logL(noisy) = −1290 ± 41; CV-logL(ideal) = −1290 ± 41) and marginalizing confidence models (model 3; CV-logL(noisy) = −1290 ± 41; CV-logL(ideal) = −1290 ± 41). Fitting only incorrect orientation discrimination trials showed that, among these observers, Type-2 False-Alarm rates were best predicted by the hierarchical visibility model, with no clear difference between the noisy and ideal version (model 7; CV-logL(noisy) = −563 ± 24; CV-logL(ideal) = −563 ± 25; Figure 5C–D).

## DISCUSSION

In this study, we designed a bias-free 2IFC task to test whether healthy volunteers can discriminate the direction of Vernier offsets while remaining unaware of the offsets themselves. Indeed, participants showed indications of unconscious perception for unmasked stimuli equal or shorter than 1150 *μ*s. We leave open the possibility that masked Verniers with ISI = 16.7 ms were processed without awareness, but in this regard our evidence was inconclusive. Nonetheless, behavior in these conditions did not satisfy our criteria demanding no metacognitive sensitivity in the absence of awareness. Overall, we found behavioral indications of unconscious perception, and yet, no conclusive evidence thereof.

Our Bayesian observer analyses further showed that models with suboptimal access to sensory evidence provided better predictions than optimal observers. In other words, we could better account for our data by simulating an observer that has degraded access to the information it uses for discriminating Vernier offsets. However, this advantage was relatively small, especially for the best fitting models (model 3 for the masking experiment, model 7 for the Tachistoscope experiment). This is consistent with the limited behavioral evidence we found for unconscious perception. Importantly, by fitting our observer models separately to easier and harder conditions, we confirmed that deviations from ideal performance indeed reflected suboptimal access to sensory evidence and not just other forms of noise.

In this study, we followed Rajananda et al. (2020) and Elosegi et al. (2023) in designing a 2IFC-based paradigm in which participants compare feature (i.e., offset) present and feature-absent intervals. This allowed us to probe awareness of the task-relevant feature (Michel, 2023), departing from the work of Peters and Lau (2015). Since Peters and Lau (2015) had participants compare stimulus-present and stimulus-absent intervals, their task is best suited to evaluate perception in the absence of *any* awareness of the stimulus. As such, the approach we adopted here constitutes a less demanding test of unconscious perception, because participants were just required to be unaware of the task-relevant feature.

In this study, we found indications that observers can be unconscious of a given feature of a stimulus while remaining able to discriminate said feature above chance. Interestingly, this contrasts with the results from Rajananda et al. (2020), who modified Peters and Lau’s (2015) 2IFC approach to probe awareness of the task-relevant feature in a facial emotion perception task, where each trial contained an emotional face and a neutral face. In contrast with Elosegi et al. (2023) and our work, they found no evidence of unconscious perception. We speculate that this might be due to the complexity of face stimuli, where multiple sub-features might be used to infer emotional content. If this is the case, making participants unconscious of the task-relevant feature might have required keeping them simultaneously unaware of all sub-features. This is potentially more demanding than having to keep them unaware of just one feature, as is the case for our Vernier offsets. And while Elosegi et al. (2023) used complex stimuli, extracting the dominant category in a stream of images depends mainly on a feedforward sweep of visual processing that is often thought to occur unconsciously (Ahissar & Hochstein, 2004; Lamme, 2015; Mashour et al., 2020). This process might thereby be preserved even when the complex task-relevant features are masked or otherwise rendered unconscious. A direct prediction is that we should be able to find other cases of unconscious ensemble perception (see Sekimoto and Motoyoshi (2022) for a promising case).

Here, using brief unmasked stimuli provided the most suggestive evidence of discrimination without awareness. We argue that this stems from the exceptional 1 *μ*s temporal resolution of our Tachistoscope (Beauny et al., 2020), which allowed a more fine-grained exploration of the dimension along which stimulus visibility was manipulated (i.e., presentation duration). Other methods rely on the temporal interval between a stimulus and a mask, which is often manipulated in steps of more than 10ms (as is the case here), or on the contrast of a masked stimulus (Peters & Lau, 2015), which is restricted by the color resolution of standard monitors.

Let us now address a couple limitations of our design. First, an important assumption of paradigms like ours is that successful performance on the subjective 2IFC task depends on the conscious perception of task-relevant features. But this assumption has been questioned (Berger & Mylopoulos, 2019). Observers could unconsciously identify the offset-present interval, which would increase performance on the 2IFC task. To the extent that our 2IFC-based visibility task shares similarities with 2IFC detection tasks, the hypothesis that healthy participants can perform the visibility 2IFC task unconsciously could be supported by reports of unconscious 2IFC detection in blindsight patients (Azzopardi & Cowey, 1997; Sahraie et al., 2010; but see Phillips, 2021, and Michel & Lau, 2021 for a response). For this reason, we emphasize that our 2IFC-based task provides a conservative estimate of unconscious perception. The goal is to strengthen the validity of the positive evidence coming from studies like this one or that of Elosegi et al. (2023).

Another downside is that our measure of participants’ awareness of the offset might have been artificially lowered by three factors. The first is response noise. Participants might be aware of the offset but erroneously report the offset-absent interval. However, we note that response noise applies to the discrimination response too, such that it would have equally impacted objective discrimination and subjective 2IFC performance. However, it is still possible that additional response noise might independently impact the Type-2 decision (Bang et al., 2019; Shekhar & Rahnev, 2021).

Secondly, even though 2IFC reports can be considered bias-free (Green & Swets, 1966; Macmillan & Creelman, 2004; Mamassian, 2020; but see Yeshurun et al., 2008 for contrasting evidence), participants might be applying a criterion for deciding when to use the sampled evidence to make an informed visibility judgment, instead of reporting a random interval. When the evidence does not surpass this criterion, participants might disengage from the task, as a way to save effort during difficult trials. As a result, participants might be conscious of the Vernier offset but still respond as if they were not. This is a concern especially for longer experiments, like ours. However, as the same issue would also affect the orientation discrimination task, we believe it had little or no impact on our results.

A third possible source of 2IFC errors is memory failures, by which observers might consciously experience the offset in one interval and then forget which interval it was by the time they are asked to report it (see Fu et al. (2023) for a review on attribute amnesia). In order to determine the effect of memory failures, we separately analyzed 2IFC performance for trials in which the task-relevant feature was presented in the first, and second interval. If participants are subject to attribute amnesia, performance on the 2IFC task should be lower when the task-relevant feature is in the first interval. This is not what our results suggest. In addition, it is important to note that having additional time for the Type-2 decision might have the opposite effect of artificially inflating performance on the 2IFC task. Indeed, several studies indicate that evidence continues to accumulate after the Type-1 decision, which might sometimes increase Type-2 accuracy relative to Type-1 accuracy (e.g., Moran et al., 2015; Murphy et al., 2015; Pleskac & Busemeyer, 2010).

Another limitation is that participants could have systematically hallucinated the presence of an offset in offset-absent intervals at the lowest durations/ISIs. If observers were in fact comparing the conscious perception of an offset in one interval to the conscious hallucination of an offset in the other interval, subjective 2IFC performance would be artificially lowered. This possibility is reinforced by the fact that observers expected to see an offset in every interval (Lin & Murray, 2014; Mack et al., 2016; Meijs et al., 2019; Pinto et al., 2015). Evaluating the impact of this confounder from behavior alone is hard. However, our Bayesian observer models do account for the possibility that an offset in the OA interval might feel more visible than an offset in the OP interval. Yet, our data was best accounted for by models producing suboptimal subjective judgements.

Before concluding, let us comment on our Bayesian observer models. Here we report the first model based on 2D-SDT (King & Dehaene, 2014; Macmillan & Creelman, 2004) that simulates visibility judgements in a 2IFC task. We made three variants of this observer and one of them (model 7) could account well for behavior relative to both masked and unmasked stimuli. Interestingly, this is a hierarchical version that computes the most-likely evidence strength of the stimulus as a lower-order process and uses this information to discriminate the offset orientation and to select the OP interval (its non-hierarchical counterpart, model 6, performed clearly worse). Another model was similarly good at recapitulating our data: a non-hierarchical observer that produces confidence judgements (model 3). Despite making very different use of their sensory evidence, models 3 and 7 produce very similar predictions of task performance and metacognitive sensitivity. Their predictions differ only for offset discrimination accuracies close to ceiling performance. Unfortunately, our datasets did not allow the exploration of this accuracy range. However, note that both share the same configuration of the evidence space, which was specifically designed to simultaneously account for the overall stimulus visibility and for the visibility of one of its features. Our hope is that these models could aid in the design of experiments that probe the relationship between these two levels of visibility.

In conclusion, we used a bias-free subjective task to uncover some indications of featural blindsight. We showed that healthy participants might be capable of performance without awareness. Even though we could not provide strong conclusions about unconscious perception, these results are a crucial step in validating the use of 2IFC tasks to probe subjective experience in consciousness studies. Further, we developed a series of Bayesian observer models, including one that simulates visibility judgements, which could guide future experimental design.

## FUNDING INFORMATION

PA was supported by an F.R.S.-FNRS Research Project T003821F (40003221) to AC. MM was supported by the Fondation Université libre de Bruxelles. SG was supported by an F.R.S.-FNRS Grant (40000378). MP was supported by a Canadian Institute for Advanced Research Fellowship in the Brain, Mind, & Consciousness Program. AC is a research director with the F.R.S.-FNRS (Belgium) and a fellow of the Canadian Institute for Advanced Research (Brain, Mind & Consciousness program). This work was partially supported by ERC AdG Grant #101055060 “EXPERIENCE” to Axel Cleeremans.

## AUTHORS CONTRIBUTIONS

PA, MM and SG equally contributed to this work. Therefore, they shall be jointly recognized as first authors of this work. Study conception and design: MM, SG. Data collection: MM, SG. Analysis and interpretation of results: PA, SG, MM, MP. Manuscript preparation: PA, AC, MM, SG, MP.

## DATA AVAILABILITY STATEMENT

The behavioral data and analysis scripts supporting the findings of this study are available in an OSF repository at the following link: https://osf.io/tbcfr/.

## Supplementary Material


