# Supplementary material for: Unconscious Perception of Vernier Offsets
Source: Open Mind (Camb). 2024 Jun 4;8:739–65. doi: 10.1162/opmi_a_00145 (PMC11185422; doi:10.1162/opmi_a_00145)
Supplement: Supplementary file 1 [file opmi-08-739-s001.docx]

**Appendix A**

**Detailed results of behavioral analyses and model fitting (BIC scores)**

**Supplementary Table 1**

*Cross-subject mean performances, Bayes Factors (BF_10_) and relative scaling factors (sf) for stimuli masked with different inter-stimulus intervals (ISIs).*

|  | **16.7 ms** | **33.3 ms** | **50.0 ms** | **66.7 ms** | **83.3 ms** | **100 ms** |
| --- | --- | --- | --- | --- | --- | --- |
| Orientation Pc | 0.580 ± 0.023 | 0.649 ± 0.035 | 0.723 ± 0.040 | 0.793 ± 0.035 | 0.812 ± 0.030 | 0.855 ± 0.031 |
| BF_10_ | 12.0 | 47.9 | 59.1 | 54.6 | 62.5 | 98.6 |
| sf | 0.074 | 0.111 | 0.146 | 0.156 | 0.178 | 0.250 |
| Interval Pc | 0.508 ± 0.015 | 0.539 ± 0.024 | 0.595 ± 0.028 | 0.657 ± 0.029 | 0.661 ± 0.034 | 0.746 ± 0.023 |
| BF_10_ | 0.66 | 7.44 | 47.8 | 83.0 | 206 | 354 |
| Sf | 0.020 | 0.048 | 0.078 | 0.081 | 0.123 | 0.250 |
| Orientation Pc (OP 1st) | 0.591 ± 0.030 | 0.648 ± 0.038 | 0.705 ± 0.037 | 0.771 ± 0.034 | 0.809 ± 0.039 | 0.862 ± 0.032 |
| Orientation Pc (OP 2nd) | 0.570 ± 0.018 | 0.650 ± 0.038 | 0.740 ± 0.047 | 0.814 ± 0.037 | 0.815 ± 0.025 | 0.849 ± 0.032 |
| BF_10_(difference) | 0.40 | 0.45 | 0.49 | 0.51 | 0.50 | 0.48 |
| sf(difference) | 0.1 | 0.1 | 0.1 | 0.1 | 0.1 | 0.1 |
| Interval Pc (OP 1st) | 0.527 ± 0.029 | 0.493 ± 0.032 | 0.572 ± 0.036 | 0.628 ± 0.042 | 0.643 ± 0.047 | 0.716 ± 0.032 |
| Interval Pc (OP 2nd) | 0.488 ± 0.038 | 0.586 ± 0.038 | 0.618 ± 0.040 | 0.686 ± 0.040 | 0.679 ± 0.043 | 0.777 ± 0.037 |
| BF_10_(difference) | 0.44 | 0.53 | 0.64 | 0.74 | 0.80 | 0.84 |
| sf(difference) | 0.1 | 0.1 | 0.1 | 0.1 | 0.1 | 0.1 |
| (Orientation Pc \| OP) | 0.581 ± 0.036 | 0.708 ± 0.037 | 0.776 ± 0.047 | 0.837 ± 0.039 | 0.871 ± 0.028 | 0.889 ± 0.029 |
| (Orientation Pc \| OA) | 0.578 ± 0.016 | 0.578 ± 0.032 | 0.639 ± 0.029 | 0.695 ± 0.036 | 0.700 ± 0.043 | 0.760 ± 0.036 |
| BF_10_(difference) | 1.24 | 2.83 | 3.81 | 3.94 | 4.03 | 4.01 |
| sf(difference) | 0.065 | 0.065 | 0.065 | 0.065 | 0.065 | 0.065 |
| T2 Hit rate | 0.506 ± 0.022 | 0.588 ± 0.025 | 0.637 ± 0.031 | 0.695 ± 0.035 | 0.709 ± 0.033 | 0.776 ± 0.021 |
| T2 False-Alarm rate | 0.497 ± 0.025 | 0.435 ± 0.018 | 0.037 | 0.418 ± 0.057 | 0.373 ± 0.052 | 0.543 ± 0.034 |
| BF_10_(difference) | 2.75 | 83.4 | 388 | 598 | 383 | 951 |
| sf(difference) | 0.077 | 0.102 | 0.117 | 0.117 | 0.117 | 0.500 |

*Note.* All performances are reported with standard errors of the mean.

**Supplementary Table 2**

*Cross-subject mean performances, Bayes Factors (BF_10_) and relative scaling factors (sf) for unmasked stimuli shown for different durations.*

|  | **980 μs** | **1150 μs** | **1349 μs** | **1583 μs** | **1857 μs** | **2179 μs** | **2557 μs** | **3000 μs** |
| --- | --- | --- | --- | --- | --- | --- | --- | --- |
| Orientation Pc | 0.551 ± 0.014 | 0.574 ± 0.013 | 0.629 ± 0.019 | 0.670 ± 0.029 | 0.732 ± 0.026 | 0.764 ± 0.018 | 0.807 ± 0.029 | 0.839 ± 0.019 |
| BF_10_ | 6.94 | 23.0 | 91.9 | 227 | 571 | 1616 | 1.1x10^5 | 5766 |
| Sf | 0.037 | 0.065 | 0.085 | 0.116 | 0.132 | 0.154 | 0.169 | 0.250 |
| Interval Pc | 0.468 ± 0.009 | 0.479 ± 0.016 | 0.494 ± 0.017 | 0.517 ± 0.018 | 0.545 ± 0.016 | 0.585 ± 0.013 | 0.628 ± 0.014 | 0.649 ± 0.021 |
| BF_10_ | 0.26 | 0.30 | 0.91 | 5.40 | 112 | 1379 | 4919 | 2.8  x10^7 |
| Sf | 0.009 | 0.009 | 0.009 | 0.022 | 0.043 | 0.064 | 0.074 | 0.250 |
| Orientation Pc (OP 1st) | 0.554 ± 0.022 | 0.565 ± 0.017 | 0.631 ± 0.024 | 0.668 ± 0.033 | 0.729 ± 0.025 | 0.764 ± 0.016 | 0.822 ± 0.028 | 0.827 ± 0.019 |
| Orientation Pc (OP 2nd) | 0.547 ± 0.023 | 0.583 ± 0.018 | 0.628 ± 0.017 | 0.672 ± 0.031 | 0.735 ± 0.031 | 0.764 ± 0.028 | 0.792 ± 0.033 | 0.850 ± 0.021 |
| BF_10_(difference) | 0.29 | 0.29 | 0.30 | 0.32 | 0.33 | 0.34 | 0.33 | 0.31 |
| sf(difference) | 0.1 | 0.1 | 0.1 | 0.1 | 0.1 | 0.1 | 0.1 | 0.1 |
| Interval Pc (OP 1st) | 0.576 ± 0.026 | 0.547 ± 0.018 | 0.533 ± 0.025 | 0.534 ± 0.030 | 0.554 ± 0.027 | 0.585 ± 0.029 | 0.635 ± 0.032 | 0.636 ± 0.032 |
| Interval Pc (OP 2nd) | 0.360 ± 0.025 | 0.410 ± 0.031 | 0.455 ± 0.036 | 0.501 ± 0.024 | 0.536 ± 0.037 | 0.586 ± 0.037 | 0.622 ± 0.022 | 0.661 ± 0.034 |
| BF_10_(difference) | 22.4 | 10.6 | 4.29 | 1.71 | 0.78 | 0.49 | 0.51 | 0.70 |
| sf(difference) | 0.1 | 0.1 | 0.1 | 0.1 | 0.1 | 0.1 | 0.1 | 0.1 |
| (Orientation Pc \| OP) | 0.561 ± 0.021 | 0.619 ± 0.019 | 0.679 ± 0.024 | 0.724 ± 0.030 | 0.809 ± 0.028 | 0.836 ± 0.021 | 0.870 ± 0.024 | 0.904 ± 0.014 |
| (Orientation Pc \| OA) | 0.541 ± 0.020 | 0.533 ± 0.013 | 0.580 ± 0.020 | 0.612 ± 0.031 | 0.643 ± 0.027 | 0.658 ± 0.021 | 0.705 ± 0.040 | 0.718 ± 0.029 |
| BF_10_(difference) | 2.40 | 4.94 | 10.1 | 24.5 | 43.6 | 60.0 | 105 | 132 |
| sf(difference) | 0.043 | 0.050 | 0.056 | 0.082 | 0.082 | 0.082 | 0.093 | 0.500 |
| T2 Hit rate | 0.477 ± 0.017 | 0.515 ± 0.017 | 0.533 ± 0.020 | 0.560 ± 0.019 | 0.601 ± 0.015 | 0.641 ± 0.017 | 0.679 ± 0.012 | 0.700 ± 0.020 |
| T2 False-Alarm rate | 0.458 ± 0.019 | 0.427 ± 0.021 | 0.425 ± 0.025 | 0.429 ± 0.019 | 0.378 ± 0.029 | 0.389 ± 0.028 | 0.413 ± 0.036 | 0.381 ± 0.024 |
| BF_10_(difference) | 21.0 | 789 | 5.7  x10^5 | 1.7  x10^15 | 1.5  x10^15 | 8.6  x10^14 | 2.9  x10^15 | 2.0  x10^15 |
| sf(difference) | 0.044 | 0.054 | 0.065 | 0.111 | 0.126 | 0.133 | 0.159 | 0.500 |

*Note.* All performances are reported with standard errors of the mean.

**Supplementary Table 3**

*Bayesian Information Criterion scores (BIC) for each Bayesian observer model, relative to the task with masked stimuli.*

|  | |  | **Full dataset** | | **Incorrect orientation**  **discrimination trials** | |
| --- | --- | --- | --- | --- | --- | --- |
| **Model** | **Best-fitting** $\boldsymbol{\sigma}_{\boldsymbol{D}}$ | | **Ideal** | **Noisy** | **Ideal** | **Noisy** |
| 1 | 0.06 ± 0.04 | | **1976 ± 96** | 1981 ± 97 | **885 ± 70** | 894 ± 69 |
| 2 | 0.35 ± 0.05 | | 2010 ± 80 | **1976 ± 96** | **894 ± 68** | 899 ± 69 |
| 3 | 0.02 ± 0.02 | | **1976 ± 95** | 1981 ± 97 | **886 ± 69** | 895 ± 69 |
| 4 | 0.14 ± 0.11 | | **2011 ± 95** | 2016 ± 95 | **892 ± 70** | 900 ± 70 |
| 5 | 0.77 ± 0.41 | | 1985 ± 86 | **1979 ± 95** | **886 ± 68** | 895 ± 68 |
| 6 | 0.86 ± 0.20 | | **1986 ± 96** | 1990 ± 95 | **892 ± 68** | 900 ± 69 |
| 7 | 0.53 ± 0.26 | | **1973 ± 96** | 1978 ± 96 | **890 ± 69** | 898 ± 70 |

*Note.* BIC values are reported as cross-subject means alongside the standard error of the mean. For each model we report the BIC for the ideal observer ($\sigma_{D}$ = 0) and the BIC for the noisy observer ($\sigma_{D}$ as free parameter). Both measures were calculated once by fitting the full dataset and once by fitting only to incorrect orientation discrimination trials. The highest score between the ideal and noisy version of each model is written in bold. Best-fitting $\sigma_{D}$s for the noisy observers are also reported.

**Supplementary Table 4**

*Bayesian Information Criterion scores (BIC) for each Bayesian observer model, relative to the task with unmasked stimuli.*

|  | |  | **Full dataset** | | **Incorrect orientation**  **discrimination trials** | |
| --- | --- | --- | --- | --- | --- | --- |
| **Model** | **Best-fitting** $\boldsymbol{\sigma}_{\boldsymbol{D}}$ | | **Ideal** | **Noisy** | **Ideal** | **Noisy** |
| 1 | 0.04 ± 0.02 | | **2759 ± 73** | 2763 ± 74 | **1267 ± 49** | 1277 ± 49 |
| 2 | 0.55 ± 0.04 | | 2827 ± 67 | **2774 ± 74** | **1283 ± 50** | **1283 ± 47** |
| 3 | 0.01 ± 0.01 | | **2759 ± 73** | 2764 ± 74 | **1266 ± 49** | 1277 ± 49 |
| 4 | 0.05 ± 0.01 | | **2772 ± 73** | 2778 ± 73 | **1265 ± 49** | 1275 ± 49 |
| 5 | 1.22 ± 0.34 | | 2773 ± 71 | **2771 ± 73** | **1272 ± 48** | 1281 ± 48 |
| 6 | 1.94 ± 0.20 | | **2780 ± 73** | 2782 ± 74 | **1289 ± 49** | 1292 ± 49 |
| 7 | 0.44 ± 0.21 | | **2759 ± 73** | 2764 ± 74 | **1262 ± 48** | 1272 ± 48 |

*Note.* BIC values are reported as cross-subject means alongside the standard error of the mean. For each model we report the BIC for the ideal observer ($\sigma_{D}$ = 0) and the BIC for the noisy observer ($\sigma_{D}$ as free parameter). Both measures were calculated once by fitting the full dataset and once by fitting only to incorrect orientation discrimination trials. The highest score between the ideal and noisy version of each model is written in bold. Best-fitting $\sigma_{D}$s for the noisy observers are also reported*.*

**Supplementary Table 5**

*Cross-participant mean best-fitting* $\sigma_{D}$ *values for the easiest and hardest conditions.*

|  | **Mean best-fitting σ_D_s** | | | | | | |
| --- | --- | --- | --- | --- | --- | --- | --- |
|  | **Model attributes** | | | **Masked stimuli** | | **Unmasked stimuli** | |
| **Model** | **Non-informative signal source** | **Hierarchical or marginalizing** | **Type of judgment** | **Hard conditions** | **Easy conditions** | **Hard conditions** | **Easy conditions** |
| 1 | Origin | Marginalizing | Confidence | **0.14 ± 0.10** | (4.0 ± 2.0) x10^-3 | **0.29 ± 0.08** | 0.05 ± 0.01 |
| 2 | Origin | Hierarchical | Confidence | **0.63 ± 0.13** | 0.26 ± 0.03 | **0.62 ± 0.12** | 0.46 ± 0.06 |
| 3 | Diagonal | Marginalizing | Confidence | **0.10 ± 0.07** | (1.4 ± 1.4) x10^-3 | **0.26 ± 0.10** | (2.2 ± 0.7) x10^-3 |
| 4 | Diagonal | Hierarchical | Confidence | **0.26 ± 0.11** | 0.03 ± 0.01 | **0.28 ± 0.09** | 0.03 ± 0.02 |
| 5 | Origin | Non applicable | Visibility | **1.71 ± 1.28** | 0.22 ± 0.13 | **3.45 ± 1.11** | 1.38 ± 0.33 |
| 6 | Diagonal | Marginalizing | Visibility | **4.74 ± 1.47** | 0.76 ± 0.15 | **6.35 ± 1.16** | 1.35 ± 0.22 |
| 7 | Diagonal | Hierarchical | Visibility | **2.50 ± 1.52** | 0.11 ± 0.06 | **3.72 ± 1.16** | 0.54 ± 0.16 |

*Note.* For each model and for each task, we report the average $\sigma_{D}$ (± standard errors) from fitting to the two easiest (ISI = 83.3ms and 100ms for masked stimuli, duration = 2557µs and 3000µs for unmasked stimuli) and the two hardest (ISI = 16.7ms and 33.3ms for masked stimuli, duration = 980µs and 1150µs for unmasked stimuli) conditions. For each task, the highest mean $\sigma_{D}$is highlighted in bold. See the caption of Table 1 for information about model attributes (and Figure 2 and the Methods section for further detail).

**Appendix B**

**Analyses replication for unmasked stimuli, excluding 3 participants.**

**Supplementary Table 6**

*Cross-subject mean performances, Bayes Factors (BF_10_) and relative scaling factors (sf) for unmasked stimuli shown for different durations.*

|  | **980 μs** | **1150 μs** | **1349 μs** | **1583 μs** | **1857 μs** | **2179 μs** | **2557 μs** | **3000 μs** |
| --- | --- | --- | --- | --- | --- | --- | --- | --- |
| Orientation Pc | 0.551 ± 0.016 | 0.585 ± 0.013 | 0.638 ± 0.021 | 0.695 ± 0.030 | 0.747 ± 0.028 | 0.782 ± 0.017 | 0.826 ± 0.033 | 0.859 ± 0.018 |
| BF_10_ | 6.75 | 23.0 | 62.0 | 124 | 246 | 869 | 640 | 899 |
| S | 0.042 | 0.069 | 0.097 | 0.123 | 0.141 | 0.163 | 0.179 | 0.250 |
| Interval Pc | 0.466 ± 0.011 | 0.476 ± 0.018 | 0.495 ± 0.021 | 0.534 ± 0.016 | 0.551 ± 0.017 | 0.595 ± 0.014 | 0.632 ± 0.017 | 0.670 ± 0.018 |
| BF_10_ | 0.20 | 0.23 | 0.81 | 12.1 | 284 | 1775 | 8.7x10^6 | 6953 |
| Sf | 0.017 | 0.017 | 0.017 | 0.026 | 0.048 | 0.066 | 0.085 | 0.250 |
| Orientation Pc (OP 1st) | 0.548 ± 0.025 | 0.579 ± 0.015 | 0.634 ± 0.029 | 0.687 ± 0.034 | 0.738 ± 0.029 | 0.772 ± 0.019 | 0.838 ± 0.032 | 0.843 ± 0.020 |
| Orientation Pc (OP 2nd) | 0.553 ± 0.028 | 0.590 ± 0.021 | 0.642 ± 0.017 | 0.702 ± 0.029 | 0.755 ± 0.034 | 0.792 ± 0.025 | 0.814 ± 0.036 | 0.874 ± 0.017 |
| BF_10_(difference) | 0.33 | 0.34 | 0.35 | 0.36 | 0.36 | 0.36 | 0.35 | 0.32 |
| sf(difference) | 0.1 | 0.1 | 0.1 | 0.1 | 0.1 | 0.1 | 0.1 | 0.1 |
| Interval Pc (OP 1st) | 0.593 ± 0.026 | 0.552 ± 0.022 | 0.543 ± 0.029 | 0.546 ± 0.036 | 0.550 ± 0.031 | 0.570 ± 0.032 | 0.626 ± 0.039 | 0.651 ± 0.033 |
| Interval Pc (OP 2nd) | 0.338 ± 0.023 | 0.401 ± 0.035 | 0.447 ± 0.044 | 0.523 ± 0.021 | 0.552 ± 0.043 | 0.620 ± 0.035 | 0.637 ± 0.024 | 0.689 ± 0.034 |
| BF_10_(difference) | 42.2 | 16.9 | 5.20 | 1.74 | 0.67 | 0.45 | 0.60 | 1.15 |
| sf(difference) | 0.1 | 0.1 | 0.1 | 0.1 | 0.1 | 0.1 | 0.1 | 0.1 |
| (Orientation Pc \| OP) | 0.565 ± 0.025 | 0.634 ± 0.018 | 0.680 ± 0.030 | 0.747 ± 0.027 | 0.820 ± 0.031 | 0.851 ± 0.023 | 0.886 ± 0.026 | 0.914 ± 0.014 |
| (Orientation Pc \| OA) | 0.537 ± 0.025 | 0.540 ± 0.016 | 0.595 ± 0.020 | 0.633 ± 0.033 | 0.660 ± 0.028 | 0.675 ± 0.022 | 0.727 ± 0.045 | 0.743 ± 0.028 |
| BF_10_(difference) | 2.04 | 3.22 | 5.59 | 9.41 | 13.3 | 16.3 | 24.6 | 23.6 |
| sf(difference) | 0.043 | 0.043 | 0.057 | 0.079 | 0.079 | 0.079 | 0.086 | 0.500 |
| T2 Hit rate | 0.479 ± 0.020 | 0.516 ± 0.020 | 0.527 ± 0.024 | 0.576 ± 0.019 | 0.604 ± 0.015 | 0.648 ± 0.021 | 0.679 ± 0.015 | 0.715 ± 0.019 |
| T2 False-Alarm rate | 0.451 ± 0.023 | 0.419 ± 0.022 | 0.432 ± 0.029 | 0.440 ± 0.015 | 0.380 ± 0.033 | 0.388 ± 0.035 | 0.404 ± 0.044 | 0.398 ± 0.024 |
| BF_10_(difference) | 15.8 | 200 | 1950 | 5.0x10^4 | 2.4x10^8 | 9.7x10^14 | 4.2x10^15 | 2.4x10^15 |
| sf(difference) | 0.048 | 0.048 | 0.068 | 0.112 | 0.130 | 0.138 | 0.158 | 0.500 |

*Note.* All performances are reported with standard errors of the mean. All measures reported here were calculated after excluding all subjects whose interval selection accuracy did not improve with more visible stimuli in both tasks.

**Supplementary Table 7**

*Cross-validated log-likelihood scores (CVlogL) for each Bayesian observer model, relative to the task with unmasked stimuli, after excluding all subjects whose interval selection accuracy did not improve with more visible stimuli in both tasks.*

|  | **Full dataset** | | **Incorrect orientation discrimination trials** | |
| --- | --- | --- | --- | --- |
| **Model** | **Ideal** | **Noisy** | **Ideal** | **Noisy** |
| 1 | - 1248 ± 37 | **- 1247 ± 37** | - 539 ± 22 | **- 538 ± 22** |
| 2 | - 1284 ± 35 | **- 1253 ± 37** | - 546 ± 20 | **- 541 ± 20** |
| 3 | **- 1248 ± 37** | **- 1248 ± 37** | **- 538 ± 21** | **- 538 ± 21** |
| 4 | **- 1255 ± 38** | **- 1255 ± 38** | **- 538 ± 22** | **- 538 ± 22** |
| 5 | - 1256 ± 36 | **- 1251 ± 37** | **- 542 ± 21** | **- 542 ± 21** |
| 6 | - 1257 ± 37 | **- 1256 ± 37** | - 546 ± 20 | **- 543 ± 20** |
| 7 | - 1248 ± 37 | **- 1247 ± 37** | **- 536 ± 21** | **- 536 ± 20** |

*Note.* CVlogL values are reported as cross-subject means alongside the standard error of the mean. For each model we report the CVlogL for the ideal observer ($\sigma_{D}$ = 0) and the CVlogL for the noisy observer ($\sigma_{D}$ as free parameter). Both measures were calculated once by fitting the full dataset and once by fitting only to incorrect orientation discrimination trials. The highest score between the ideal and noisy version of each model is written in bold.

**Supplementary Table 8**

*Bayesian Information Criterion scores (BIC) for each Bayesian observer model, relative to the task with unmasked stimuli, after excluding all subjects whose interval selection accuracy did not improve with more visible stimuli in both tasks.*

|  |  | **Full dataset** | | **Incorrect orientation**  **discrimination trials** | |
| --- | --- | --- | --- | --- | --- |
| **Model** | **Best-fitting** $\boldsymbol{\sigma}_{\boldsymbol{D}}$ | **Ideal** | **Noisy** | **Ideal** | **Noisy** |
| 1 | 0.05 ± 0.02 | **2683 ± 64** | 2687 ± 65 | **1217 ± 43** | 1226 ± 43 |
| 2 | 0.55 ± 0.06 | 2758 ± 59 | **2697 ± 65** | 1232 ± 39 | **1231 ± 40** |
| 3 | 0.02 ± 0.01 | **2683 ± 64** | 2687 ± 65 | **1215 ± 42** | 1225 ± 42 |
| 4 | 0.05 ± 0.02 | **2697 ± 66** | 2703 ± 66 | **1216 ± 44** | 1225 ± 43 |
| 5 | 1.46 ± 0.37 | 2698 ± 62 | **2695 ± 64** | **1220 ± 39** | 1228 ± 40 |
| 6 | 1.76 ± 0.20 | **2703 ± 64** | 2704 ± 65 | **1233 ± 39** | 1236 ± 39 |
| 7 | 0.53 ± 0.24 | **2683 ± 64** | 2687 ± 65 | **1211 ± 41** | 1222 ± 41 |

*Note.* BIC values are reported as cross-subject means alongside the standard error of the mean. For each model we report the BIC for the ideal observer ($\sigma_{D}$ = 0) and the BIC for the noisy observer ($\sigma_{D}$ as free parameter). Both measures were calculated once by fitting the full dataset and once by fitting only to incorrect orientation discrimination trials. The highest score between the ideal and noisy version of each model is written in bold. Best-fitting $\sigma_{D}$s for the noisy observers are also reported.

**Supplementary Table 9**

*Cross-participant mean best-fitting* $\sigma_{D}$ *values for the easiest and hardest conditions, relative to the task with unmasked stimuli, after excluding all subjects whose interval selection accuracy did not improve with more visible stimuli in both tasks.*

|  | **Mean best-fitting σ_D_** | | | | |
| --- | --- | --- | --- | --- | --- |
|  | **Model attributes** | | | **Unmasked stimuli** | |
| **Model** | **Non-informative signal source** | **Hierarchical or marginalizing** | **Type of judgment** | **Hard conditions** | **Easy conditions** |
| 1 | Origin | Marginalizing | Confidence | **0.27 ± 0.10** | 0.06 ± 0.02 |
| 2 | Origin | Hierarchical | Confidence | **0.65 ± 0.13** | 0.49 ± 0.06 |
| 3 | Diagonal | Marginalizing | Confidence | **0.29 ± 0.12** | (2.2 ± 0.8) x10^-3 |
| 4 | Diagonal | Hierarchical | Confidence | **0.24 ± 0.08** | (1.4 ± 0.5) x10^-3 |
| 5 | Origin | Non applicable | Visibility | **3.91 ± 1.33** | 1.63 ± 0.36 |
| 6 | Diagonal | Marginalizing | Visibility | **7.16 ± 1.24** | 1.19 ± 0.20 |
| 7 | Diagonal | Hierarchical | Visibility | **4.09 ± 1.42** | 0.63 ± 0.19 |

*Note.* For each model and for each task, we report the average $\sigma_{D}$ (± standard errors) from fitting to the two easiest (ISI = 83.3ms and 100ms for masked stimuli, duration = 2557µs and 3000µs for unmasked stimuli) and the two hardest (ISI = 16.7ms and 33.3ms for masked stimuli, duration = 980µs and 1150µs for unmasked stimuli) conditions. For each task, the highest mean $\sigma_{D}$is highlighted in bold. See the caption of Table 1 for information about model attributes (and Figure 2 and the Methods section for further detail).
